# Auxin response factors (ARFs) differentially regulate rice antiviral immune response against rice dwarf virus

**DOI:** 10.1371/journal.ppat.1009118

**Published:** 2020-12-02

**Authors:** Qingqing Qin, Guangyao Li, Lian Jin, Yu Huang, Yu Wang, Chunhong Wei, Zhihong Xu, Zhirui Yang, Haiyang Wang, Yi Li

**Affiliations:** 1 The State Key Laboratory of Protein and Plant Gene Research, School of Life Sciences, Peking University, Beijing, China; 2 Department of Biology, Southern University of Science and Technology, Shenzhen, China; 3 State Key Laboratory for Conservation and Utilization of Subtropical Agro-Bioresources, South China Agricultural University, Guangzhou, China; University of California, Davis Genome Center, UNITED STATES

## Abstract

There are 25 auxin response factors (ARFs) in the rice genome, which play critical roles in regulating myriad aspects of plant development, but their role (s) in host antiviral immune defense and the underneath mechanism remain largely unknown. By using the rice-*rice dwarf virus* (RDV) model system, here we report that auxin signaling enhances rice defense against RDV infection. In turn, RDV infection triggers increased auxin biosynthesis and accumulation in rice, and that treatment with exogenous auxin reduces OsIAA10 protein level, thereby unleashing a group of OsIAA10-interacting OsARFs to mediate downstream antiviral responses. Strikingly, our genetic data showed that loss-of-function mutants of *osarf12* or *osarf16* exhibit reduced resistance whereas *osarf11* mutants display enhanced resistance to RDV. In turn, OsARF12 activates the down-stream *OsWRKY13* expression through direct binding to its promoter, loss-of-function mutants of *oswrky13* exhibit reduced resistance. These results demonstrated that OsARF 11, 12 and 16 differentially regulate rice antiviral defense. Together with our previous discovery that the viral P2 protein stabilizes OsIAA10 protein via thwarting its interaction with OsTIR1 to enhance viral infection and pathogenesis, our results reveal a novel auxin-IAA10-ARFs-mediated signaling mechanism employed by rice and RDV for defense and counter defense responses.

## Author summary

The phytohormone auxin is often critical for plant growth and orchestrates many developmental processes. Here we find that rice accumulates more auxin upon RDV infection and treatment with exogenous auxin enhances rice tolerance to RDV infection. Auxin treatment reduces the protein level of OsIAA10, thus releasing a group of OsIAA10-interacting OsARFs to mediate downstream antiviral responses. Among the 25 ARFs in the rice genome, their functions on regulation of rice antiviral defense are diversified. Our findings elucidate a novel auxin-OsIAA10-ARFs-mediated signaling mechanism employed by rice and RDV for defense and counter defense responses. These findings significantly deepen our understanding of virus-host interactions and provide novel targets for molecular breeding (or engineering) rice cultivars resistant to RDV.

## Introduction

Rice is a major staple crop feeding more than half of world’s population [1]. Viral infection causes enormous losses in rice yield and quality, posing a constant threat to global food security [1–3]. Breeding of viral resistant rice cultivars is an effective and environmentally friendly means to meet this challenge, yet such effort has been hampered by our limited understanding of the mechanisms underneath antiviral responses in rice [1, 2, 4, 5]. *Rice dwarf virus* (RDV), a member of the genus *Phytoreovirus* in the family *Reoviridae* transmitted by leafhoppers (*Nephotettix cincticeps*), is a major threat to rice production in Asia [6–9]. The genome of RDV is composed of 12 double strand RNA segments (*S1*-*S12*). Among them, *S1*, *S2*, *S3*, *S5*, *S7*, *S8* and *S9* encode structural proteins, while *S4*, *S6*, *S10*, *S11* and *S12* encode nonstructural proteins of RDV [6, 7, 10, 11]. RDV infection disturbs the normal physiology and metabolism of rice, leading to dwarfism, production of excess tillers, dark green leaves with white chlorotic specks, delayed maturation, higher rate of abortive grains, and consequently reduced grain yield with deteriorated quality [12–15].

Plants have evolved multiple defense mechanisms to combat with the continuous threat of viral infection [1, 2, 4, 16–20]. As a counter-defense, plant viruses also have evolved strategies to manipulate plant responses (such as plant’s hormone responses) for their own benefit [4, 12–14, 18, 21–23]. For example, it has been shown that virus can modulate a number of plant hormone signaling pathways (such as gibberellin, ethylene and auxin) to counteract the host plant’s defense responses [11–14, 23–29]. Previous studies in our laboratory have shown that RDV-encoded Pns11 protein promotes ethylene production to enhance plant susceptibility to viral infection [13]. In addition, we showed that the viral P2 protein interacts with *β-ent*-kaureen oxidases to reduce gibberellic acid synthesis, resulting in dwarfism [14]. P2 also contributes to the dwarf phenotype of infected rice plants by interfering with auxin signaling through interacting with OsIAA10, thus enhancing viral infection and pathogenesis [12]. Most recently, it was reported that several different plant RNA viruses manipulate rice auxin signaling by targeting OsARF17, one of the components of auxin signaling pathway to facilitate infection [29]. These studies demonstrate the broad significance of viral inhibition or manipulation of multiple hormonal pathways to benefit infection and enhance disease symptoms [11–14, 27–29].

Auxin is an endogenous hormone that plays an important role in regulating cell division, expansion, and differentiation, thus controlling many aspects of plant growth and development [30–36]. It has been established that perception of auxin by its receptor protein TIR1 activates the auxin signaling pathway, which triggers the degradation of IAA proteins, a family of repressors of auxin signaling, thus unleashing a family of ARF transcription factors from the inhibitory effect of IAA proteins to regulate downstream auxin-responsive gene expression and ultimately auxin responses [31–39]. We previously showed that the RDV P2 protein specifically interacts with domain II of OsIAA10 protein and thwarts the interaction of OsIAA10 with OsTIR1, thereby interfering with the host plant’s auxin signaling by preventing the degradation of OsIAA10 and increased susceptibility to RDV [12]. However, the downstream signaling mechanism of rice susceptibility to RDV infection upon OsIAA10 accumulating still remains unknown.

In this study, we investigated the mechanism by which auxin signaling modulates RDV resistance in rice. We found that RDV infection causes increased auxin synthesis and accumulation, and that auxin treatment reduces the protein level of OsIAA10, thus releasing its interacting partner OsARFs to activate the downstream genes. Loss-of-function mutants of either *osarf12* or *osarf16* exhibit reduced resistance whereas *osarf11* or *osarf5* confers enhanced resistance to RDV. Thus, these OsARFs appear to play different roles in the RDV defenses. Our results reveal a novel auxin-IAA10-ARFs-mediated signaling mechanism employed by rice and RDV for defense and counter defense responses. These findings significantly deepen our understanding of virus-host interactions and provide novel targets for molecular breeding (or engineering) rice cultivars against RDV.

## Results

### Exogenous auxin treatment enhances rice tolerance to RDV infection via down-regulating OsIAA10

**Fig 1:**
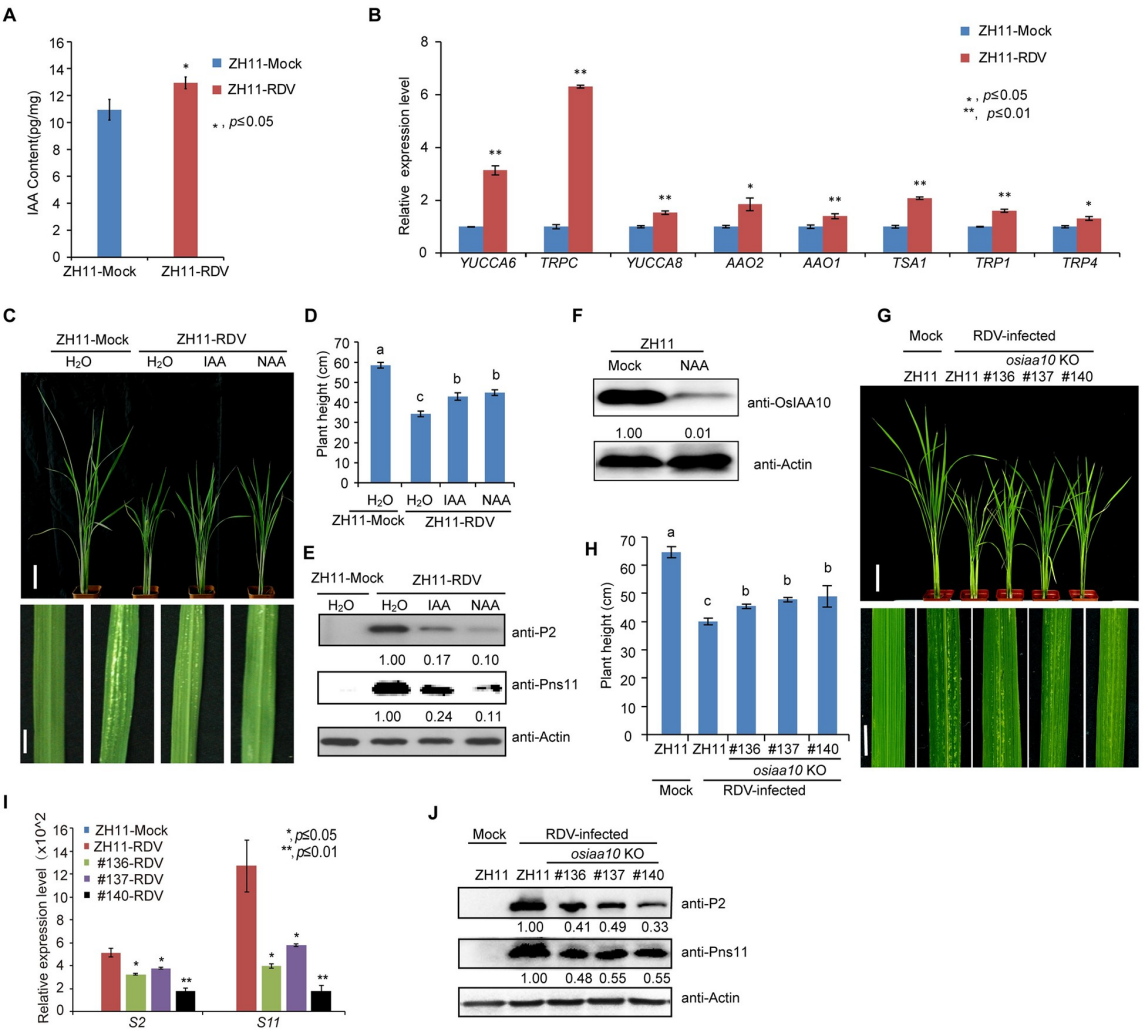
Exogenous auxin treatment enhances rice tolerance to RDV infection via down-regulating OsIAA10. (A) IAA content is higher in RDV-infected plants. ZH11-Mock, uninfected ZH11 plants, ZH11-RDV, RDV-infected ZH11. (B) Increased expression of some auxin biosynthesis genes in RDV-infected rice. ZH11-Mock, uninfected ZH11 plants, ZH11-RDV, RDV-infected ZH11. (C) Phenotypes of RDV-infected ZH11 rice plants pretreated with H_2_O, IAA or NAA, respectively. Photos were taken at four-week-post-inoculation (wpi). Scale bars, 10 cm (upper panel) and 1 cm (lower panel). (D) Schematic representation of plant height for the plants in (C). The average (±SD) values were obtained from three biological repeats. Different letters indicate significant difference (p< 0.05) based on the Tukey-Kramer HSD test. (E) Western blots showing the accumulation of RDV proteins in the corresponding rice lines shown in (C). Actin was used as a loading control. (F) Western blots showing the accumulation of OsIAA10 protein after auxin treatment and in the ZH11 (Mock). Actin was used as a loading control. (G) RDV-infected WT (ZH11) and *osiaa10* KO rice plants. Photos were taken at 4 weeks after RDV-inoculation. The sizes of white specks on the leaves represent the degree of disease symptoms. Scale bars, 10 cm (upper panel) and 1 cm (lower panel). **(H)** Schematic representation of plant height for the plants in (G). The average (±SD) values were obtained from three biological repeats. Different letters indicate significant difference (p< 0.05) based on the Tukey-Kramer HSD test. (I) qRT-PCR showing the accumulation of RDV genomic RNAs in the corresponding rice lines shown in (G). (J) Western blots showing the accumulation of RDV proteins in the corresponding rice lines in (G). Actin was used as a loading control.

We previously showed that RDV infection stabilizes OsIAA10 protein and that knocking down *OsIAA10* expression in rice causes enhanced resistance to RDV infection [12], suggesting that auxin signaling plays a positive role in rice antiviral defense. To test this further, we measured the IAA content in healthy and RDV infected rice, and found that the auxin accumulation is higher in RDV-infected rice (Fig 1A). qRT-PCR analysis showed that many auxin biosynthesis genes were up-regulated in RDV-infected rice, such as *YUCCA6*, *TRPC*, *YUCCA8*, *AAO2*, *AAO1*, *TSA1*, *TRP1*, and *TRP4* (Fig 1B). These results suggest that high level IAA in rice plays critical roles in defense against RDV infection. Then we treated rice seedlings by pre-culturing them in liquid medium supplemented with auxin (IAA and NAA) before inoculation with viruliferous leafhopper. The results showed that IAA or NAA treatment successfully inhibited crown root elongation (S1A Fig) without deterring leafhopper infestation (S1 Table). However, NAA or IAA pretreatment dramatically attenuated disease symptoms caused by RDV infection, as exemplified by less dwarfism and fewer chlorotic specks compared to the control rice plants (Fig 1C and 1D). Consistent with the observed phenotypes, accumulation of RDV encoded proteins (Fig 1E) was reduced in the NAA or IAA pretreated rice plants. The disease incidence was also reduced by pre-treatment with NAA or IAA (S1B Fig and S2 Table). Similarly, spraying IAA also effectively enhanced the resistance of rice seedlings against RDV (S1D Fig and S2 Table). qRT-PCR analysis showed that the expression of two representative auxin-responsive genes, *OsIAA10* and *OsGH3*.*2*, was significantly induced shortly after IAA spraying (S1C Fig). In addition, western blot analysis showed that the level of OsIAA10 protein was decreased significantly after NAA treatment (Fig 1F). These results indicate that external application of auxin enhances rice antiviral defense to RDV infection.

We previously showed that knocking down *OsIAA10* expression in rice causes enhanced resistance to RDV infection [12], suggesting that OsIAA10 negatively regulates rice response to RDV infection. To verify this notion, we generated *OsIAA10* knockout (KO) lines using the CRISPR/Cas9 technology. Three independent *osiaa10* KO rice lines (KO#136, KO#137 and KO#140) with mutations at different codons in the coding region were obtained (S2A–S2D Fig). We then inoculated rice seedlings of these rice mutant lines and wild type (WT) rice plants with RDV using viruliferous leafhoppers. Consistent with the previous report [12], we found that at four weeks post inoculation (4 wpi), the *osiaa10* KO lines exhibited much weaker disease symptoms compared to the WT (ZH11) plants, such as less dwarfism, fewer tillers and chlorotic flecks (Fig 1G and 1H). The disease incidence was also lower in the *osiaa10* KO plants than in the WT (ZH11) plants (S2E Fig and S2 Table). Consistently, the accumulation of both RDV genomic RNAs and proteins was less in the RDV infected *osiaa10* KO rice plants than those in the infected WT (ZH11) plants (Fig 1I and 1J). These results convincingly demonstrate that OsIAA10 plays a negative role in rice antiviral defense against RDV infection.

### OsIAA10 interacts with a group of OsARFs proteins

**Fig 2:**
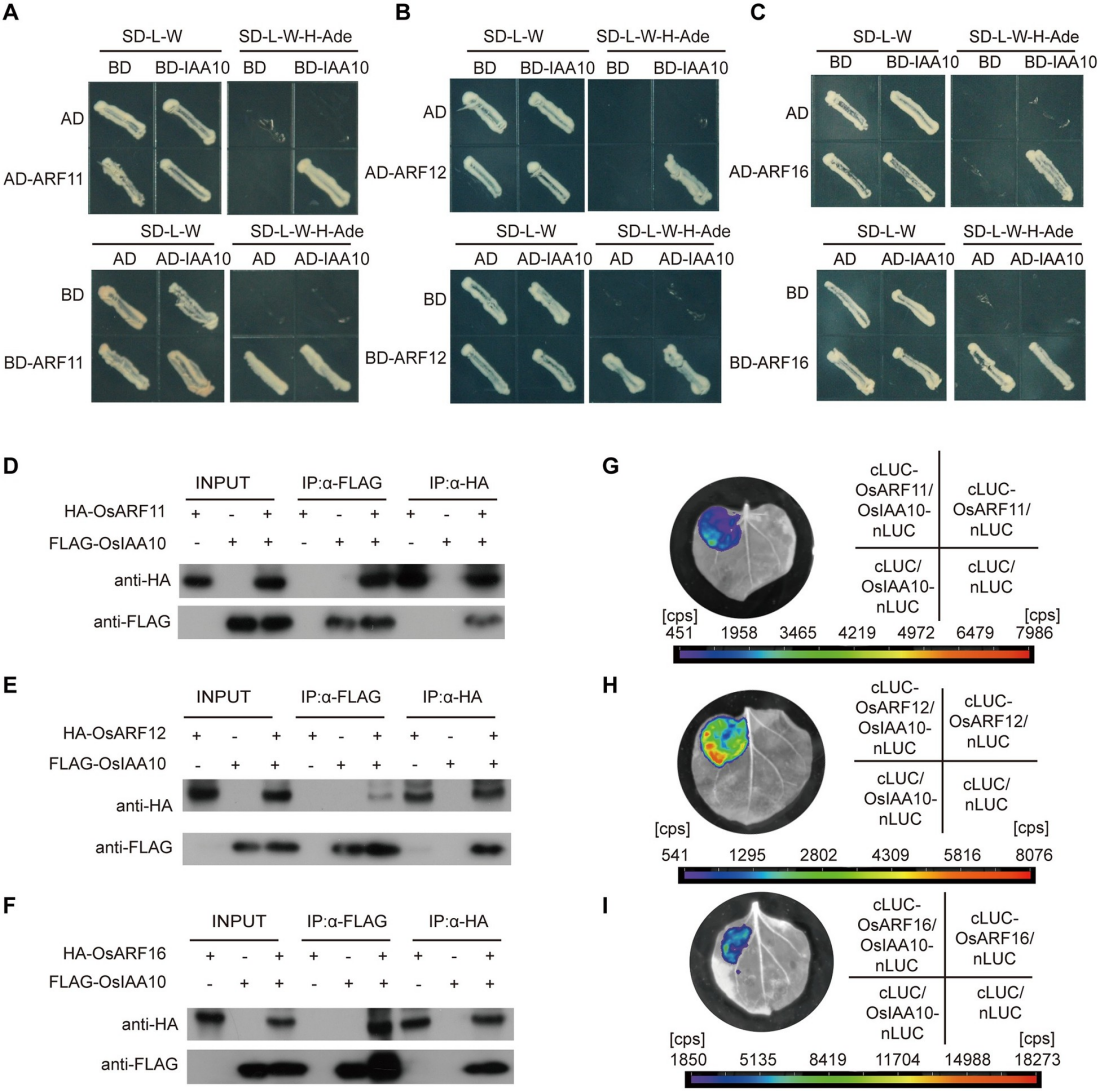
OsIAA10 interacts with OsARF11, 12, 16 in plant. (A) Yeast two-hybrid assay confirming the OsARF11 and OsIAA10 interaction. (B) Yeast two-hybrid assay confirming the OsARF12 and OsIAA10 interaction. (C) Yeast two-hybrid assay confirming the OsARF16 and OsIAA10 interaction. (D) Co-immunoprecipitation confirmed the interaction between OsIAA10 and OsARF11. (E) Co-immunoprecipitation confirmed the interaction between OsIAA10 and OsARF12. (F) Co-immunoprecipitation confirmed the interaction between OsIAA10 and OsARF16. (G) LCI assay showed the interaction between OsIAA10 and OsARF11 in plants. (H) LCI assay showed the interaction between OsIAA10 and OsARF12 in plants. (I) LCI assay showed the interaction between OsIAA10 and OsARF16 in plants.

As repressors of auxin signaling pathways, Aux/IAA family proteins regulate auxin-responsive gene expression through interacting with and inhibiting the activity of the ARF transcription factors [40–42]. There are 25 *OsARF* genes in the rice genome, we cloned all these 25 *OsARF* genes and tested the interactions of OsIAA10 with these 25 OsARFs (S3A Fig). To further explore the downstream factors regulated by OsIAA10, we firstly conducted a yeast two hybrid (Y2H) assay to identify the interacting OsARFs of OsIAA10. Y2H assay showed that several OsARFs, including 5, 6, 11, 12, 16, 17, 19, 21 and 25, interacted with OsIAA10 (Fig 2A–2C and S3B Fig). In order to confirm the interactions in plants, we carried out co-immunoprecipitation (Co-IP) assays through transiently expressing FLAG (synthetic octapeptide)-tagged OsIAA10 and hemagglutinin (HA)- tagged OsARFs in tobacco (*Nicotiana benthamiana*). Results showed that HA-OsARF11, HA-OsARF12, HA-OsARF16, HA-OsARF19 and HA-OsARF21 were coimmunoprecipitated with FLAG-OsIAA10 in the co-expressing samples (Fig 2D–2F and S4G Fig). To further test these interactions *in vivo*, we conducted a firefly luciferase (LUC) complementation imaging assays (LCI Assay). Luminescence signals were only detected in cLUC-OsARF11, cLUC-OsARF12, cLUC-OsARF16, cLUC-OsARF19, cLUC-OsARF21 and OsIAA10-nLUC co-expressing regions but not in the negative controls, while cLUC-OsARF5, cLUC-OsARF6, cLUC-OsARF17, cLUC-OsARF25 and OsIAA10-nLUC co-expressing regions showed no signals as well as the negative controls (Fig 2G–2I and S4 Fig). Taken together, these data establish that OsIAA10 interacts with OsARF11, OsARF12, OsARF16, OsARF19 and OsARF21 in plants, which indicate that these OsARFs may be functioned downstream of OsIAA10 during rice antiviral defense against RDV infection.

### Antiviral functions of OsARFs are diversified

**Fig 3:**
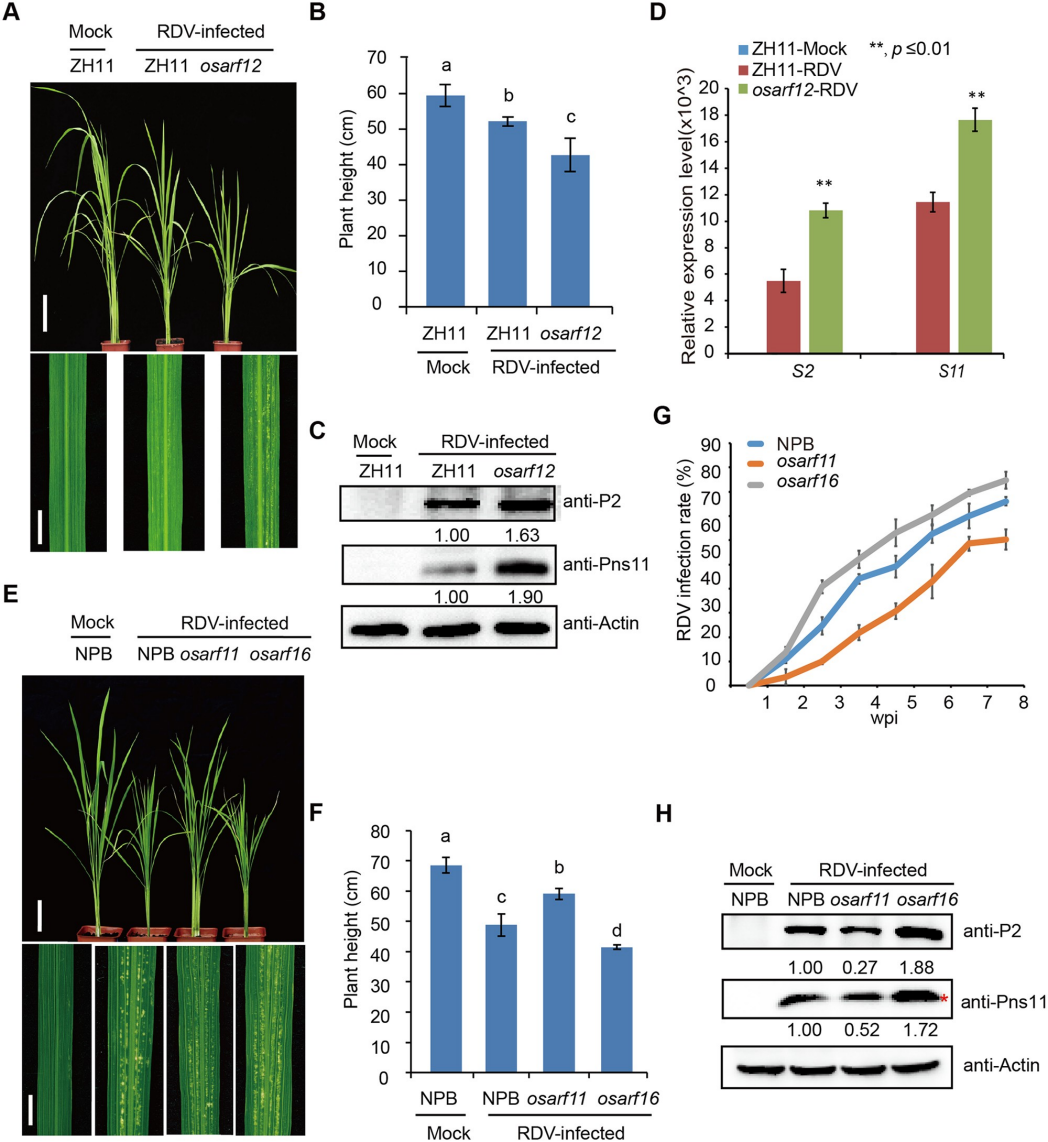
Loss-of-function mutants of *osarf12* or *osarf16* exhibit reduced resistance whereas *osarf11* mutants display enhanced resistance to RDV. (A) Phenotypes of RDV-infected WT (ZH11) and *osarf12* mutants. Photos were taken at 4 weeks after RDV-inoculation. The sizes of white specks on the leaves represent the degree of disease symptoms. Scale bars, 10 cm (upper panel) and 1 cm (lower panel). (B) Schematic representation of plant height for the plants in (A). The average (±SD) values were obtained from three biological repeats. Different letters indicate significant difference (p< 0.05) based on the Tukey-Kramer HSD test. (C) Accumulation of RDV proteins in the corresponding lines. Actin was used as a loading control for proteins. (D) Accumulation of RDV RNAs in the corresponding lines. The average (±SD) values were obtained from three biological repeats. The error bars indicate SD. (E) Phenotypes of RDV-infected WT (NPB), *osarf11* and *osarf16* mutants. Photos were taken at 4 weeks after RDV-inoculation. The sizes of white specks on the leaves represent the degree of disease symptoms. Scale bars, 10 cm (upper panel) and 1 cm (lower panel). (F) Schematic representation of plant height for the plants in (E). The average (±SD) values were obtained from three biological repeats. Different letters indicate significant difference (p< 0.05) based on the Tukey-Kramer HSD test. (G) RDV infection rates of *osarf11*, *osarf16* and WT (NPB) from one wpi to eight wpi. Inoculation assays were repeated three times. The error bars indicate SD. (H) Accumulation of RDV proteins in the corresponding lines. Actin was used as a loading control for proteins. “*” indicated the RDV Pns11 protein.

**Fig 4:**
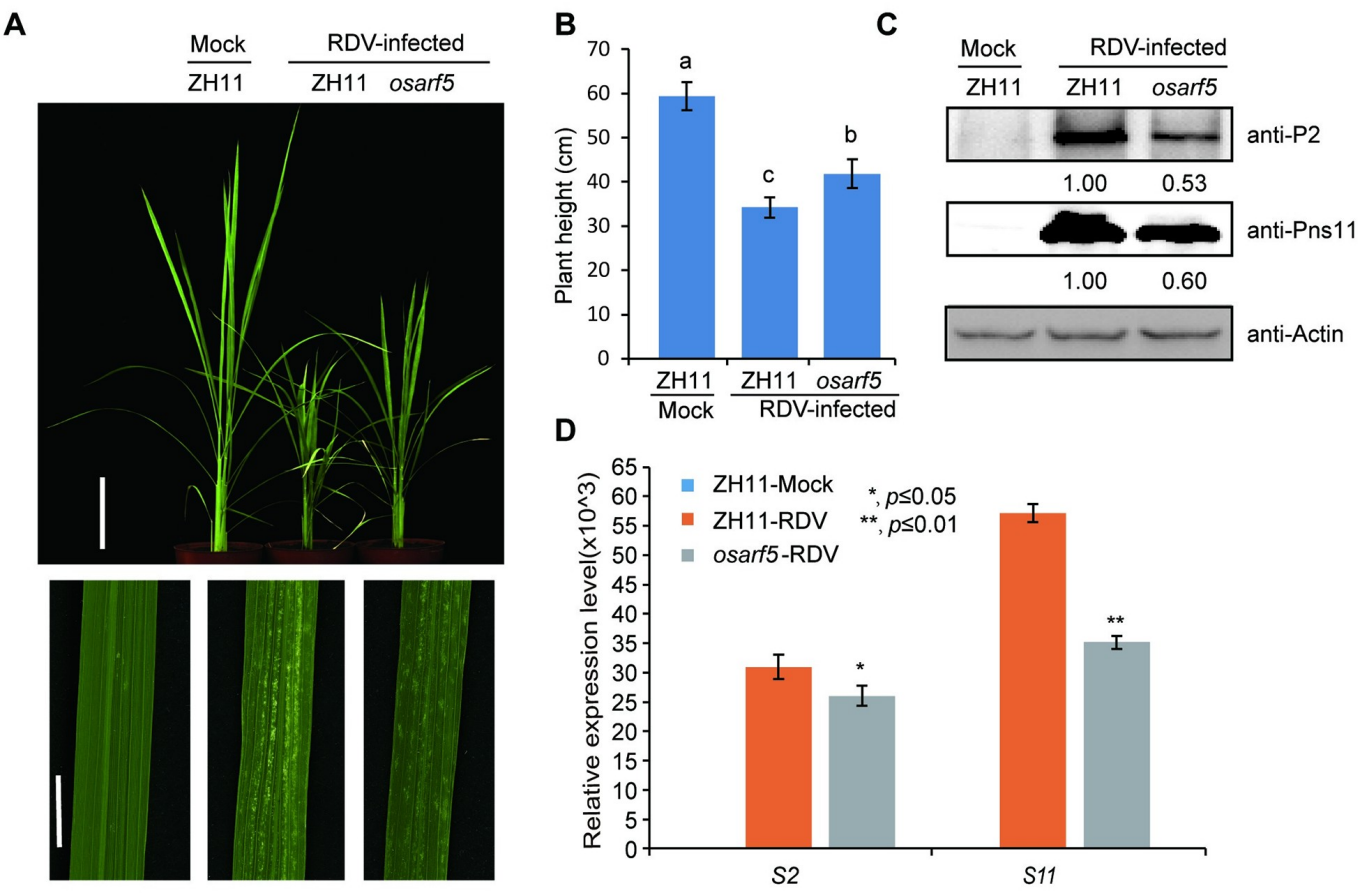
Loss-of-function *osarf5* mutants are more resistant to RDV. (A) Phenotypes of RDV-infected WT (ZH11) and *osarf5* mutant. Photos were taken at 4 weeks after RDV-inoculation. The sizes of white specks on the leaves represent the degree of disease symptoms. Scale bars, 10 cm (upper panel) and 1 cm (lower panel). (B) Schematic representation of plant height for the plants in (A). The average (±SD) values were obtained from three biological repeats. Different letters indicate significant difference (p< 0.05) based on the Tukey-Kramer HSD test. (C) Accumulation of RDV proteins in the corresponding lines. Actin was used as a loading control for proteins. (D) Accumulation of RDV RNAs in the corresponding lines. The average (±SD) values were obtained from three biological repeats. The error bars indicate SD.

We next tested whether these OsARFs are involved in rice resistance against RDV infection. Among the 25 OsARFs, some of them are predicted to possess activation activity, in which the middle domains are enriched in glutamine, while others possess repression activity, in which the repressor middle domains are enriched in proline, serine and threonine [43–49]. The IAA10-interacting OsARFs including 11, 12, 16, 19 and 21 are all transcriptional activators [43, 44]. So, we selected OsARF11, OsARF12 and OsARF16, which located on different branches of the ARF family phylogenetic tree (S3A Fig), for functional analyses. OsARF5, which does not interact with IAA10 in our assays and also belongs to the activation group, was used as a control. We obtained *Tos17*-insertion mutant lines for *OsARF5* (RMD_04Z11AZ68), *OsARF11* (ND6043), *OsARF12* (RMD_ATosR-04Z11AG72) and *OsARF16* (NC6645) from SALK (http://signal.salk.edu/cgi-bin/RiceGE) (S5 Fig). RT-PCR results showed that *Tos17* insertions in the *OsARF11*, *12* and *16* genes disrupted their expression in the mutants (S5 Fig). We inoculated each mutant line using viruliferous leafhoppers. As expected, the *osarf12* and *osarf16* mutants showed severer symptoms, higher disease incident, and higher accumulation of RDV proteins than the WT control plants at 4 wpi (Fig 3 and S2 Table). However, the *osarf11* and *osarf5* mutants showed much more tolerance or enhanced resistance to RDV infection and lower disease incident, and lower accumulation of RDV proteins than the WT control plants at 4 wpi (Figs 3 and 4 and S2 Table). These results suggest that *OsARF12* and *OsARF16* positively regulate whereas *OsARF11* and *OsARF5* negatively regulate rice resistance against RDV infection.

**Fig 5:**
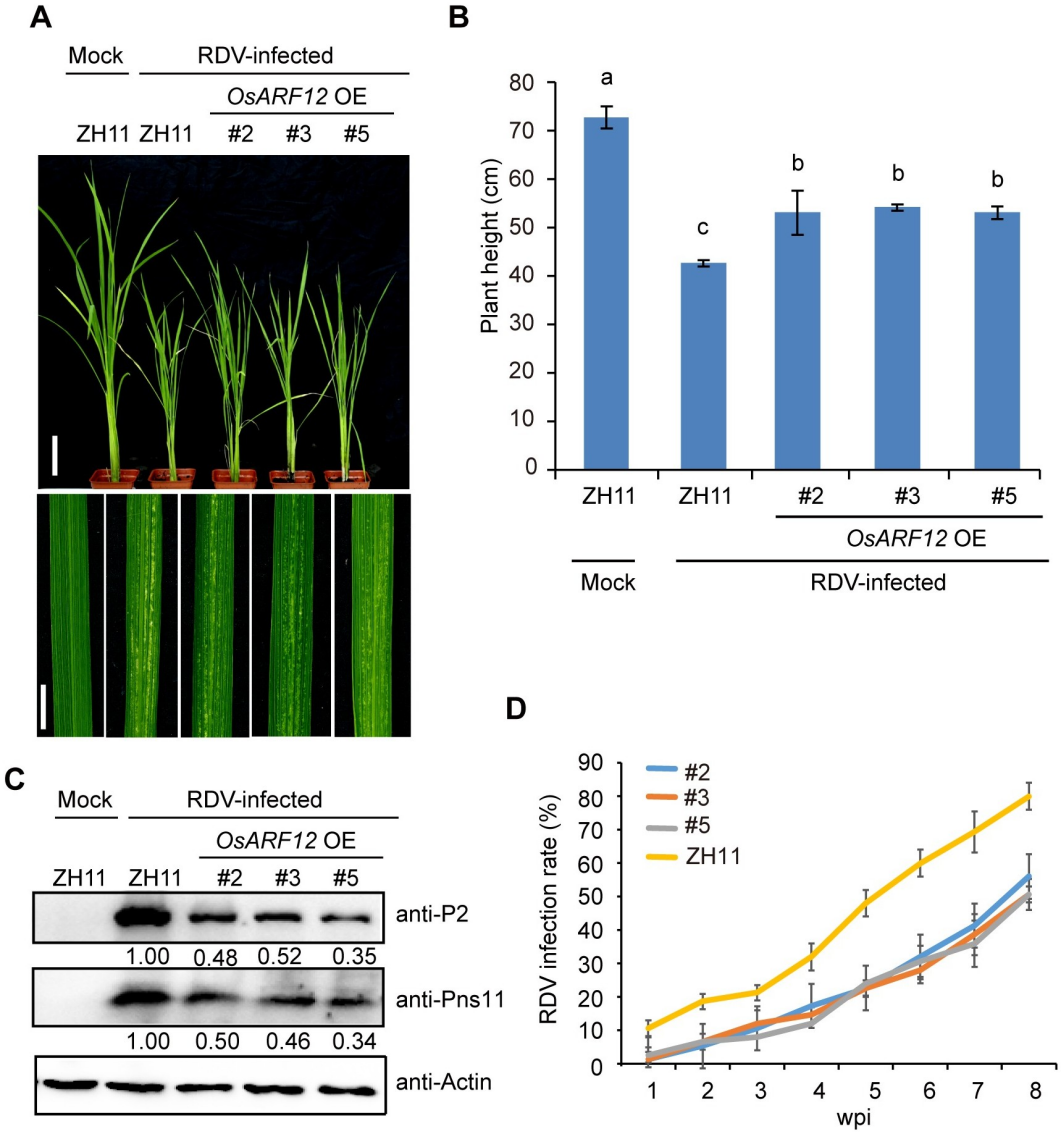
*OsARF12* OE plants exhibit enhanced resistance to RDV infection. (A) Phenotypes of RDV-infected WT (ZH11) and *OsARF12* OE lines. Photos were taken at 4 weeks after RDV-inoculation. The sizes of white specks on the leaves represent the degree of disease symptoms. Scale bars, 10 cm (upper panel) and 1 cm (lower panel). (B) Schematic representation of plant height for the plants in (A). The average (±SD) values were obtained from three biological repeats. Different letters indicate significant difference (p< 0.05) based on the Tukey-Kramer HSD test. (C) Western blots showing the accumulation of RDV proteins in the corresponding lines. Actin was used as a loading control for proteins. (D) RDV infection rates of the corresponding lines from one wpi to eight wpi. Inoculation assays were repeated three times. The error bars indicate SD.

In order to broaden our understanding of the developmental expression and tissue specificity of the interacting ARFs, we tested the tissue and developmental expression patterns of *OsARF5*, *OsARF11*, *OsARF12* and *OsARF16* using reverse transcriptase-PCR. Among these four *OsARFs*, *OsARF11* only expressed in specific tissue, such as high level in stem, lower level in root and not detectable in other tissues, indicating that *OsARF11* might influence virus systemically transmission (S6A and S6B Fig). All of these four *OsARFs* had the highest expression level during 2–3 weeks post sowing, the expression level of *OsARF12* was always higher than other three *OsARFs* (S6A and S6B Fig). To test if *OsARF12* may also play a role in virus defense, we also generated *OsARF12* overexpression (OE) plants in the ZH11 background. Three positive *OsARF12* OE transgenic lines (#2, #3, and #5) were chosen for the virus infection assay (S7 Fig). We inoculated the *OsARF12* OE transgenic lines and WT (ZH11) seedlings using viruliferous leafhoppers (25 seedlings were used for each line) (Fig 5A). At 4 wpi, all three *OsARF12* OE lines displayed weaker stunting symptoms and fewer chlorotic flecks compared with the WT (ZH11) control plants (Fig 5A and 5B), suggesting that the *OsARF12* OE seedlings were more tolerant to RDV infection compared with the WT (ZH11) plants. Consistently, the RDV-infected *OsARF12* OE rice plants had lower disease incidence and viral protein levels than RDV-infected WT (ZH11) plants (Fig 5C and 5D and S2 Table).

To further substantiate the role of *OsARF12* in viral resistance, we also generated *osarf12* knockout (KO) lines using the CRISPR/Cas9 technology in the ZH11 background. Three independent *osarf12* KO rice lines (*osarf12* KO#1, KO#5 and KO#6) with mutations at different codons in the coding sequence of *OsARF12* were obtained (S8A Fig). Virus infection assay showed that in contrast to the *OsARF12* OE lines, all three lines of *osarf12* KO mutants showed severer phenotypes, such as severer dwarfism and more specks, compared to the WT (ZH11) rice plants (S8B and S8C Fig). The percentage of infected plants as well as the accumulation of RDV encoded protein were also higher in the RDV infected *osarf12* KO than those in WT (ZH11) seedlings (S8D–S8F Fig, and S2 Table). These results suggest that knockout of *OsARF12* causes reduced resistance against RDV in rice.

### The possible targets regulated by the OsARFs and their function in antiviral defense

**Fig 6:**
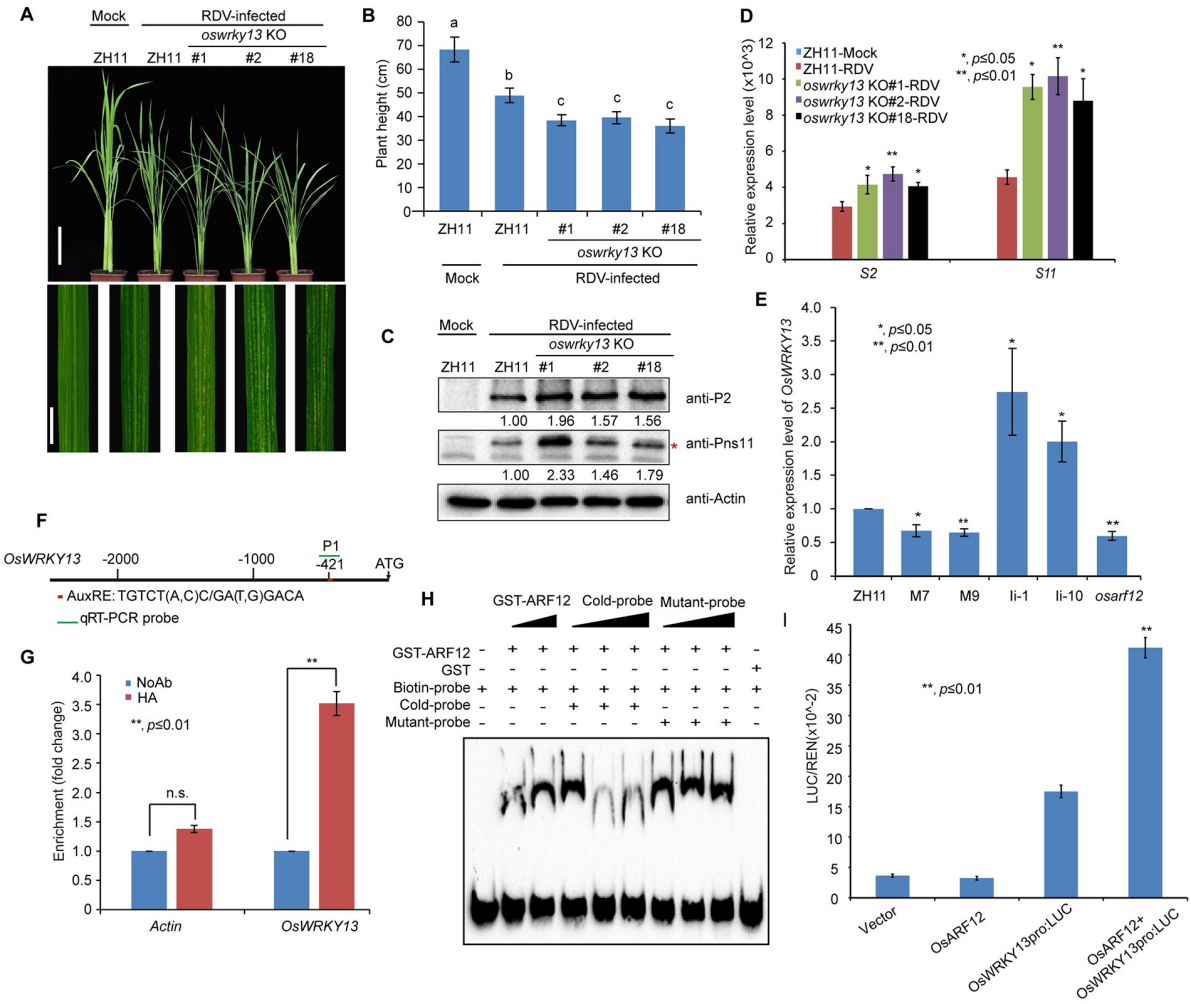
*OsWRKY13* is a possible target of OsARF12, OsARF12 binds the promoter of *OsWRKY13*, *oswrky13* KO are more susceptible to RDV infection. (A) Symptoms of RDV-infected WT (ZH11) and *oswrky13* KO lines. Photos were taken at 4 weeks after RDV-inoculation. The sizes of white specks on the leaves represent the degree of disease symptoms. Scale bars, 10 cm (upper panel) and 1 cm (lower panel). (B) Schematic representation of plant height for the plants in (A). The average (±SD) values were obtained from three biological repeats. Different letters indicate significant difference (p< 0.05) based on the Tukey-Kramer HSD test. (C) Accumulation of RDV proteins in the corresponding lines. Actin was used as a loading control for proteins. “*” indicated the product of RDV Pns11 protein. (D) Accumulation of RDV RNAs in the corresponding lines. The average (±SD) values were obtained from three biological repeats. The error bars indicate SD. (E) qRT-PCR analysis of *OsWRKY13* expression in ZH11, M7, M9, Ii-1, Ii-10, and *osarf12* mutant rice seedlings. M7 and M9 are transgenic rice lines overexpressing OsIAA10P116L. Ii-1 and Ii-10 are OsIAA10 RNAi transgenic rice lines. Expression levels were normalized against the values obtained for *OsEF1a*. The value obtained from WT (ZH11) plants was arbitrarily set at 1.0. The average (±SD) values were obtained from three biological repeats. The error bars indicate SD. (F) Sequence analysis of the auxin response element (AuxRE) in the *OsWRKY13* promoter. AuxRE sequence: TGTCT (A, C) C/ GA (T, G) GACA indicated by red box. PCR primers are indicated by the green line. (G) ChIP-qPCR assay shows that OsARF12 binds to the *OsWRKY13* promoter region containing the AuxRE element. NoAb, no antibody. HA, HA antibody. Actin as a negative control. (H) EMSA shows that the OsARF12 DNA binding domain binds to the AuxRE element of the *OsWRKY13* promoter. The biotinylated probe containing the AuxRE sequence was incubated with GST-OsARF12 (121–247 aa), while the probes incubated with no protein or GST protein were used as negative controls. Non-labeled probes were used as the cold competitors. The probe sequence is TGGTTCGTGATTAAGGGTTTGGTTACACCGTGTCCCGCTCACGGATAGGCTGCTTAATTCTCTTT; Mutant probe: GTTACACCGAAAAAAGCTCACGGAT. (I) Ratio of firefly luciferase (LUC) to Renilla luciferase activity in rice protoplasts co-transformed with different reporter and effector construct combinations. Error bars represent standard deviations of three biological replicates run in triplicate. **P < 0.01 according to Student’s t-test.

Because OsARF12 and 16 positively regulate rice antiviral defense, we hypothesized that these two ARFs may positively regulate some antiviral resistance genes. We next asked which target genes are activated by these two OsARFs to participate in rice resistance against RDV infection. Previously, we found that several defense response genes, such as *OsPR2*, *OsPR10*, *OsJAZ12*, *OsWRKY13* and *OsWRKY45*, were down-regulated in the OsIAA10-stablized rice plants, which were more sensitive to RDV infection [12]. At the same time, we conducted RNA sequencing analysis of *OsIAA10*-related rice plants, *OsIAA10* RNAi #1 rice line and *OsIAA10P116L*-overexpressing M7 rice line [12], because we wanted to screen the functional pathways downstream of *OsIAA10*. We found that both up-regulated genes in *OsIAA10* RNAi line and down-regulated genes in *OsIAA10P116L*-overexpressing M7 rice line were enriched in many signaling pathways, include SA signaling pathway (S9 Fig). Therefore, we further confirmed the expression patterns of genes involved in SA signaling pathway in these indicated rice plants (S10 Fig). We found that the SA signaling pathway was activated in the *osiaa10* KO rice lines and *OsARF12* OE lines (S10A and S10C Fig), but repressed in the *osarf12* and *osarf16* mutant rice plants (S10D and S10E Fig). The expression pattern of these genes in *osarf5* and *osarf11* were variable (S10B and S10E Fig), which might due to the function of *OsARF5* and *OsARF11* are different from *OsIAA10*-regualted immune response during virus infection. Given that SA signaling induced PR genes expression always correlated with the accumulation of active oxygen species (ROS) [50, 51], we also detected the ROS levels in these rice lines (S11 Fig). Consistently, ROS accumulated to higher levels in the *osiaa10* KO and *OsARF12* OE rice plants and lower levels in the *osarf12* compared to WT plants. Notably, it has been shown that overexpression of *OsWRKY13* confers enhanced resistance to bacterial blight and fungal blast in rice [52]. But the antiviral function of *OsWRKY13* is unknown. Given that *OsWRKY13* is involved in the regulations of genes in SA and JA signaling, we further tested whether *OsWRKY13* is a downstream target gene of *OsARF12* in regulating antiviral defense against RDV in rice. We generated *OsWRKY13* knockout (*oswrky13* KO) rice lines. *oswrky13* KO lines were generated using the CRISPR/Cas9 technology. Three independent *oswrky13* KO lines with different mutations were chosen for RDV infection assay (S13 Fig). We inoculated the seedlings of the above rice lines and WT (ZH11) seedlings with RDV (Fig 6A). Consistent with our hypothesis, the *oswrky13* KO plants displayed a more severe stunting symptom and more chlorotic flecks than the WT (ZH11) control plants (Fig 6A and 6B). Consistently, accumulation of both genomic RNAs and RDV proteins was significantly increased in the *oswrky13* KO plants compared to the WT (ZH11) plants (Fig 6C and 6D). These results support the notion that *OsWRKY13* plays a positive role in enhancing rice antiviral defense against RDV infection.

To test whether *OsWRKY13* is regulated by the OsARFs, we firstly used qRT-PCR analysis to examine the expression levels of *OsWRKY13* in WT (ZH11), *OsIAA10P116LOE* (in which the highly conserved proline residue at position 116 was changed to leucine to create an auxin-induced degradation resistant OsIAA10 mutant protein), OsIAA10RNAi (Ii-1 and Ii-10) rice seedlings [12]. The results showed that the expression level of *OsWRKY13* was increased in the *OsIAA10* RNAi rice lines (Ii-1 and Ii-10) and the *osiaa10* KO rice lines (#136, #137, #140), but decreased in the *OsIAA10P116LOE* rice lines M7 and M9, compared to the WT (ZH11) (Fig 6E and S12 Fig). We then measure the expression level of *OsWRKY13* in the mutants of *osarf12*. The results showed that the expression level of *OsWRKY13* in the mutants of *osarf12* was reduced (Fig 6E). We obtained similar results in the *osarf12* KO (#1, #5, #6) rice lines (S12A Fig). By contrast, the expression level of *OsWRKY13* showed no significant changes in the *osarf5*, *osarf11* and *osarf16* mutants (S12B Fig). These results suggest that *OsWRKY13* may be a downstream target gene subject to regulation by the OsIAA10-OsARF12 module.

To investigate whether *OsWRKY13* is a direct target of OsARF12, we performed chromatin immunoprecipitation (ChIP)-qPCR and EMSA assays. Sequence analysis of auxin response elements (AuxRE) on *OsWRKY13* promoter indicated that there is one AuxRE element in the *OsWRKY13* promoter (Fig 6F). ChIP-qPCR assays showed that the fragment (at position from 333 bp to 524 bp) containing the AuxRE element in the *OsWRKY13* promoter was highly enriched in the anti-HA-immunoprecipitated chromatin from the HA-OsARF12 transgenic plant, but not in the control (NoAb.), demonstrating a direct binding of HA-OsARF12 to the promoter sequence of *OsWRKY13* (Fig 6G). To confirm this, we generated the OsARF12 DNA binding domain (121-247aa) recombinant protein (fused to glutathione S-transferase [GST]). Purified GST-OsARF12 fusion protein was incubated with biotin-labeled probes containing either wild-type AuxRE sequence or mutated AuxRE sequence of the *OsWRKY13* promoter, with or without competitor sequences, and then subjected to electrophoretic mobility shift assay (EMSA). The gel-shift assay showed that the GST-OsARF12 fusion protein interacted with the probe containing the wild type AuxRE sequence but not the probe containing mutated AuxRE sequence (Fig 6H), thus confirming a specific interaction between the OsARF12 protein with the promoter of *OsWRKY13*. Further, we performed a transient transfection assay to examine the intrinsic transcription regulatory activity of OsARF12 on *OsWRKY13* expression. The results showed that OsARF12 protein was able to activate the luciferase reporter genes driven by the *OsWRKY13* promoter in rice protoplasts (Fig 6I). Together, these results indicate that OsARF12 regulates the expression of *OsWRKY13* by directly binding to the AuxRE element in its promoter (Fig 6H).

## Discussion

Accumulating evidence suggests that many viruses maximize their multiplication through disrupting the host plant’s auxin signaling [12, 27–29]. For example, *Tobacco mosaic virus* (TMV)’s replicase protein interacts with IAA26 protein, disrupting the localization and stability of IAA26 protein, causing altered auxin signaling and reduced disease resistance in the host plant [27, 28]. Several different plant RNA viruses manipulate rice auxin signaling by using independently evolved viral proteins to target OsARF17, the key component of auxin signaling pathway to facilitate infection [29]. We previously showed that stabilization or increased accumulation of OsIAA10 enhances rice susceptibility to RDV infection. However, the exact molecular mechanisms underlying auxin signaling-mediated antiviral defense response have not been unraveled yet.

In this study, we showed that RDV infection triggers increased auxin synthesis and accumulation in rice, and that pretreatment with auxin can reduce OsIAA10 protein accumulation and enhance rice resistance to RDV infection (Fig 1 and S1 and S14 Figs). We further showed that OsIAA10 interacted with specific OsARFs in plants (Fig 2 and S4 Fig). Notably, we showed that antiviral functions of these OsARFs are diversified. OsARF12 or 16 knock out weakens while overexpression of *OsARF12* enhances rice resistance to RDV infection (Figs 3 and 5 and S8 Fig), indicating a positive role of OsARF12 and OsARF16 in rice defense against RDV. However, *osarf11* mutants showed increased resistance to RDV (Fig 3). Transcriptional mediation by ARFs is central to auxin response, and the opposing functions of these ARFs on gene targets create equilibrium. We also found that ARFs that do not interact with IAA10 can also affect RDV infection, such as OsARF5. RDV infection assay showed that the *osarf5* mutant showed mild disease symptoms with less viral RNAs and proteins accumulation than WT plants (Fig 4). This auxin-dependent but *IAA10*-independent pathway indicates that other *OsIAAs* or other *OsIAA*-independent pathways might participate in rice antiviral defense against RDV infection. Previously studies showed that OsARF17, which interacts with OsIAA20, could regulate rice antiviral defense against different plant RNA viruses, including *rice black-streaked dwarf virus* (RBSDV), *Southern rice black streaked dwarf virus* (SRBSDV) and *Rice stripe virus* (RSV). In addition, ARFs are regulated in diverse ways, in addition to regulation by auxin and its interacting IAA proteins, ARFs are also regulated by miRNAs at the transcriptional or post-transcriptionally levels. Therefore, due to the considerable variation in ARFs, understanding the multifaceted levels of ARF regulation and function will contribute to explaining how auxin response to the plant antiviral defense and growth.

Moreover, we demonstrated that OsARF12 directly binds to the AuxRE element in the *OsWRKY13* promoter to activate its expression (Fig 6). We further showed that *oswrky13* KO plants had decreased resistance to RDV (Fig 6). Thus, our results uncover a novel auxin-IAA10-ARF12 signaling-mediated mechanism utilized by the rice plant for defense against RDV. Intriguingly, RDV can manipulate the host plant’s auxin signaling process for counter defense. We thus envisage that RDV infection triggers increased auxin synthesis and accumulation in rice, despite higher level of free IAA content in RDV rice, the viral P2 protein stabilizes OsIAA10 protein by blocking its interaction with the SCF^TIR1/AFBs^ complex and subsequent degradation, leading to inhibition of its interactive OsARFs and reduced viral resistance. In addition, we also uncovered a novel OsARF12-OsWRKY13 regulatory module among these interacted OsARFs. These findings greatly deepen our understanding of the molecular mechanisms underlying the defense and counter defense responses between the host plants and viruses.

It should be noted that in addition to affecting auxin signaling, RDV infection also affects the signaling pathways of a number of other plant hormones, including gibberellin and ethylene [11–14]. Previous studies in our laboratory have shown that RDV-encoded Pns11 protein enhances rice susceptibility to RDV by interacting with OsSAMS1 (S-adenosyl-L-methionine synthetase), enhancing its enzymatic activity and leading to increasing production of SAM, ACC, and ethylene [13]. In addition, RDV-encoded P2 protein interacts with *β-ent*-kaureen oxidases, an enzyme in the gibberellic acid biosynthesis pathway, leading to diminished accumulation of GA and to the dwarf phenotype exhibited by RDV-infected rice plants [14]. Thus, it is apparent that RDV could hijack multiple hormone signaling pathways to benefit infection and its multiplication [12–14]. Untangling the complex cross talks between the various hormonal signaling pathways during the defense and counter defense responses between RDV and its host will be interesting avenue for future research.

It is worth mentioning that in this study, we show that auxin signaling enhances rice resistant to RDV infection through the degradation of OsIAA10 protein and subsequent release of its interacted OsARFs, and these OsARFs play different roles in antiviral process. As we all known, these ARFs are the effectors of auxin response and translate the specific chemical signal into the regulation of a defined set of genes. Given the limited number of ARFs in auxin signaling, distinct functions among the ARF family probably contribute to the multiple unique auxin responses in plant development. ARF transcription factors bind to auxin response elements in the promoter of their target genes and they have specific transcriptomes, for example, the activator OsARF11 binds to AuxRE in the promoter of the brassinosteroid receptor gene *OsBRI1* and regulates its expression to regulate plant height and leaf angle [53]. OsARF19 also controls leaf angle trough *OsGH3-5* and *OsBRI1* [54]. OsARF16 was required in Fe deficiency response and Fe uptake, but also established a critical link between auxin and -Pi response in rice [55]. These data show that there may be significant functional specialization among ARFs in rice. Data show that the spacing or orientation of auxin response element leads to different affinities for the ARFs, it may explain the functional diversity of ARFs and how different ARFs involved in different developmental processes. ARFs are regulated in diverse ways, in addition to regulation by auxin, ARFs are also regulated by miRNAs at the transcriptional or post-transcriptionally level, and the importance of non-auxin-regulated pathways is becoming more evident [56]. It is also worth highlighting that the biological functions for most ARFs are not yet clear. It will also be worthy investigating how these *OsARFs* act in timely and spatially regulated manner to orchestrate rice immune response to virus infection. In addition, whether the herein reported OsIAA10-OsARFs module is involved in defense against other plant pathogens and viruses also remains to be examined. Moreover, OsIAA and OsARF proteins are known to regulate many important processes of plant growth, development and responses to various biotic and abiotic stresses, and in many cases, through cross-talking with other signaling pathways [46–49, 53–55]. Their roles in coordinating plant growth and development versus defense responses also represent interesting avenues for future research.

## Materials and methods

### Plant growth and virus inoculation

Plant growth and RDV inoculation methods were carried out as previously described [11, 12]. Rice seedling plants cv. Zhonghua11 were grown in a greenhouse at 28–30°C under natural sunlight. Two weeks old seedlings were exposed to the RDV-carrying or virus-free (Mock) leafhoppers (2–3 insects per plant for two days). Two days after inoculation, the insects were removed, and the plants were kept in the same conditions. Then their infection rate was recorded every week. The sample was collected for viral RNA and protein test four weeks after the infestation.

### DNA constructs and rice transformation

The open reading frames (ORFs) of *OsARFs* were amplified from cDNA of ZH11, then cloned into appropriate destination vectors by recombination (Vazyme) or T4 DNA ligase (NEB). pCambia2300:35S was used for generating the overexpression construct. The generation of CRISPR/Cas9 knock out lines and *Agrobacterium tumefaciens*-mediated rice transformation were carried out in BioRun Co., Ltd. (Wuhan, China). All primers used in this study are listed in S3 and S4 Tables.

### Measurement of free IAA

For quantification of free IAA in Mock and RDV rice, whole seedlings were harvested and approximately 200 mg of fresh tissues were used for IAA extraction and measurement as previously described [57]. Three biological replicates were performed.

### Yeast two-hybrid assay

The CDS of *OsARFs* and *OsIAA10* were cloned into pGADT7 and pGBKT7 vector for Y2H. Yeast transformation was carried out according to the instructions (Clontech, Mountain View, California, USA). Yeast AH109 cells were co-transformed with *OsIAA10* and *OsARFs*. All yeast transformants were grown on an SD/-Leu/-Trp and then transferred to SD/-Leu/-Trp/-His/-Ade medium for interaction test.

### Co-Immunoprecipitation assay

The pWM101-HA-OsARFs and pCambia1301-FLAG-OsIAA10 constructs were co-infiltrated into *N*. *benthamiana* leaves by agroinfiltration. After 3 days, the samples were extracted with IP buffer [50 mM Tris-HCl pH7.5, 150 mM NaCl, 0.1% NP-40, 5 mM DTT, protease inhibitor cocktail Complete Mini tablets (Roche)] as described previously [12]. Then we transferred the supernatant of the sample to the anti HA- mAb or anti FLAG- mAb (Agarose Conjugate), and incubated at 4°C for 1 hr. After washing with wash buffer (50 mM Tris-HCl pH7.5, 150 mM NaCl) three times, 20 μl of sample buffer (50 mM Tris-HCl pH6.8, 2% SDS, 6% Glycerol, 0.1M DTT, 0.02% bromophenol blue) were added and boiled at 95°C for 10 min and then centrifuged. The samples were loaded into the SDS-PAGE gels and the OsARFs and OsIAA10 proteins were detected with the corresponding antibody.

### Firefly luciferase complementation imaging assay

The CDS of *OsIAA10* and *OsARFs* were inserted into the pCambia1300-nLUC and pCambia1300-cLUC vectors, respectively. All these constructs were transformed into the *A*. *tumefaciens* strain EHA105. Four different combinations of *A*. *tumefaciens* were co-infiltrated on the same leaves of *Nicotiana benthamiana* as shown in the figure. Three days later, 0.2 mM luciferin (Promega, USA) was infiltrated into the same positions that *A*. *tumefaciens* infiltrated, then luciferase activity was detected with a low-light cooled CCD imaging apparatus (NightOWL II LB983 with indiGO software).

### RNA extraction, semi-quantitative reverse transcription-PCR (RT-PCR) and quantitative real-time PCR (qRT-PCR)

Total RNA was extracted with TRIzol Reagent (Invitrogen) according to the manufacturer’s instructions. The total RNAs were treated with RQ1 RNase-free DNase (Promega) to remove genomic DNA and then reverse transcribed using the SuperScript III Reverse Transcriptase kit (Invitrogen) according to the manufacturer’s instructions. The cDNA was used as the template for qRT-PCR. qRT-PCR was performed using the SYBR Green Real-Time PCR Master Mix (Mei5 Biotech, Beijing, China) following the manufacturer’s instructions. The rice *OsEF1a* gene was used as an internal control. The primers are listed in S4 Table.

### Auxin treatment assays

For auxin treatment assay, two-week old rice seedlings were pre-cultured in a liquid culture solution contain 1 μM IAA or 0.1 μM NAA. After 3 days, the root phenotypes were photographed using a digital camera, then used for RDV infection. Alternatively, we sprayed the rice leaves with 50 μM IAA solution, and the samples were collected 2 hours after the spraying for gene expression test.

### RNA-seq analysis

Total RNAs were extracted from WT (ZH11) and transgenic rice lines (2 wpi, 14-d-old seedlings) using TRIzol Reagent (Invitrogen) according to the manufacturer’s instructions. The RNA-seq analyses were performed at Bionova Company. Libraries were constructed through adaptor ligation and were subjected to pair-ended sequencing with a 150-necleotide reading length. FastQC software was used to access the quality of raw sequencing reads. After removing adaptor and low-quality reads, clean reads were mapped to rice genome MSU7.0 using TopHat. Responsive genes were identified by reads per kilobase per million reads (RPKM) and edgeR software was used to identify the differential expressed genes. The multiple-testing *P*-value < 0.05 and fold change (FC >2) was used to determine whether the gene was significantly differentially expressed or not.

### Dual-luciferase reporter system

One kilobase of the *OsWRKY13* promoter was inserted into the pGreen II 0800 vector and used as a reporter construct. The coding sequence of OsARF12 was inserted into pCambia2300 and used as the effector construct. Rice protoplasts were isolated from leaf sheaths of 2-week old rice plants grown under LD conditions. The pGreen II 0800-LUC-OsWRKY13pro vector was co-transformed with pCambia2300-OsARF12 into protoplasts and incubated at 28°C overnight under weak light. The relative Luc activity (Luc/Ren ratio) was detected with the dual-luciferase reporter assay system (Promega) and a Promega GLO-MAX 20/20 microplate luminometer.

### Electrophoretic mobility shift assay

The pGEX-4T-1-ARF12 (121-247aa) construct and the empty pGEX-4T-1 vector were individually transformed into the *E*. *coli* strain Transetta (DE3) (Transgene, Beijing, China). The soluble GST fusion proteins were purified using glutathione sepharose beads (Amersham Buckinghamshire, UK). The AuxRE probes of the *OsWRKY13* promoter were labeled with biotin. An unlabeled probe was used for the competition assay. The EMSA was performed according to the Light Shift Chemiluminescent EMSA Kit (Thermo Scientific). The primers of the probes used are listed in S4 Table.

### Chromatin immunoprecipitation (ChIP)

ChIP were performed according to the method described previously [58]. 2-week old seedlings of wild type (WT) and the HA-OsARF12 overexpression rice plants were collected, and cross-linked using 1% (v/v) formaldehyde under vacuum for 8 min. Then the chromatin complexes were isolated and sonicated for ChIP by incubating with anti-HA antibody (Sigma). The DNA was eluted from the antibody-conjugated beads with the elution buffer and used for qRT-PCR. The primers of the promoters used for qRT-PCR are listed in S4 Table.

### Histochemical staining of ROS

The histochemical staining of H_2_O_2_ and O^2-^ was performed as described previously with some modifications [50, 51]. Briefly, the rice leaves were cut into 1 cm length and were infiltrated with 10 mM Tris-HCl (pH 6.5) contained 1 mg/mL 3,3′-diaminobenzidine (DAB, Sigma) or 50 mM sodium phosphate (pH 7.0) contained 0.05% nitroblue tetrazolium (NBT, Sigma), respectively, followed by incubation at 37°C in the dark for 16 hours. Then washed the leaves with bleaching solution (ethanol: acetic acid = 3:1) to bleach out the chlorophyll at 70°C. Finally, the leaves were photographed using stereoscope under uniform lighting.

## Supporting information

S1 Fig Auxin treatment analysis. (A) Elongation of crown roots is inhibited by treatment with IAA or NAA in ZH11. Photograph was taken with a Nicon camera. Scale bar, 2.5 cm; Red arrows indicate the newly grown crown roots. (B) RDV infection rates in ZH11 rice plants pretreated with auxin. Time course of RDV infection rates in rice plants pretreated with H_2_O, IAA or NAA from one to eight wpi. Inoculation assays were repeated three times. The error bars indicate SD. (C) qRT-PCR assay showing the relative expression levels of *OsIAA10* and *OsGH3*.*2* after pre-spraying with water or IAA in ZH11. *OsEF1a* was used as a reference. The average (± SD) values were obtained from three biological repeats. Significant differences (*P< 0.05, **P< 0.01) are indicated based on Students’ t-test. (D) Time course of RDV infection rates for ZH11 rice plants pre-spraying with water or IAA from one to eight wpi. Inoculation assays were repeated three times. The error bars indicate SD.
(TIF)

S2 Fig Identification and infection rates of the *osiaa10* KO transgenic lines. (A) ‘T’ insertion in the line *osiaa10* KO #136. The mutation leads to premature termination of OsIAA10. (B) 938 bp and 79 bp deletion in the line *osiaa10* KO #137, causing premature termination of OsIAA10. (C) ‘GG’ deletion in the line *osiaa10* KO #140, causing premature termination of OsIAA10. (D) Phenotypes of non-RDV infected WT (ZH11) and *osiaa10* KO lines. Photos were taken at 4 weeks, Scale bars, 10 cm. (E) RDV infection rates in the *osiaa10* KO rice plants. Time course of RDV infection rates in the *osiaa10* KO rice plants from one to eight wpi. Inoculation assays were repeated three times. The error bars indicate SD.
(TIF)

S3 Fig IAA10 interacts with several OsARFs in yeast. (A) The phylogenetic tree of OsARFs. Phylogenetic relationship among the rice OsARF proteins. The unrooted tree was generated using ClustalX program by neighbor-joining method. Bootstrap values form 100 replicates are indicated at each node. (B) Y2H screen for OsARFs that interact with OsIAA10. Yeast two-hybrid assay for confirming the OsARF and OsIAA10 interaction. The bait protein OsARF is expressed as a GAL4 DNA binding domain fusion, and the OsIAA10 is expressed as GAL4 DNA activation domain fusions in yeast AH109 cells. Positive interaction is indicated by the ability of cells to grow on medium lacking His (-H) and Adeline (-Ade). Vectors expressing the GAL4 binding domain (BD) or GAL4 activating domain (AD) are used as negative controls. SD, synthetic dropout medium; -L, lacking Leu; -W, lacking Trp.
(TIF)

S4 Fig IAA10 interacts with several OsARFs proteins. (A)There is no interaction between OsIAA10 and OsARF5. (B) There is no interaction between OsIAA10 and OsARF6. (C) There is no interaction between OsIAA10 and OsARF17. (D) OsIAA10 interacts with OsARF19 in plants. (E) OsIAA10 interacts with OsARF21 in plants. (F) There is no interaction between OsIAA10 and OsARF25. The left diagram indicates the leaf panels that were infiltrated with A. tumefaciens containing the different combinations of indicated constructs. Cps indicates signal counts per second. (G) Co-immunoprecipitation confirmed the interaction between OsIAA10 and OsARF11, OsARF12, OsARF16, OsARF19, OsARF21.
(TIF)

S5 Fig Identification of *osarf12*, *osarf11*, *osarf16* and *osarf5* mutants. (A) Tos17 insertion site in the *osarf12* mutant. The black box represents the exon, and the black line represents the intron. (B) PCR analysis confirms the integration of Tos17 in OsARF12. The lower bands indicate that Tos17 is inserted into the OsARF12 genic region in the *osarf12* mutant. (C) Semi-quantitative RT-PCR analysis shows that the *OsARF12* gene is not expressed in the mutant. *Actin* was used as a loading control. (D) Tos17 insertion site in the *osarf11* mutant. The black box represents the exon, and the black line represents the intron. (E) PCR analysis confirms the integration of Tos17 in *OsARF11*. The lower bands indicate that Tos17 is inserted into the *OsARF11* genic region in the *osarf11* mutant. (F) RT-PCR analysis showing that the *OsARF11* gene is not expressed in the mutant. Actin was used as a loading control. (G) Tos17 insertion site in the *osarf16* mutant. The black box represents the exon, and the black line represents the intron. (H) PCR analysis confirms the integration of Tos17 in *OsARF16*. The lower bands indicate that Tos17 is inserted into the *OsARF16* genomic region in the *osarf16* mutant. (I) Semi-quantitative RT-PCR analysis shows that the *OsARF16* gene is not expressed in the mutant. *Actin* was used as a loading control. (J) Tos17 insertion site in the *osarf5* mutant. The black box represents the exon, and the black line represents the intron. (K) PCR analysis confirms the integration of Tos17 in *OsARF5*. The lower bands indicate that Tos17 is inserted into the *OsARF5* genic region in the *osarf11* mutant. (L) Semi-quantitative RT-PCR analysis shows that the *OsARF5* gene is not expressed in the mutant. *Actin* was used as a loading control. (M) Phenotypes of non-RDV infected WT (ZH11) and *osarf12* mutant lines. Photos were taken at 4 weeks, Scale bars, 10 cm. (N) Phenotypes of non-RDV infected WT (NPB) and *osarf11*, *osarf16* mutant lines. Photos were taken at 4 weeks, Scale bars, 10 cm.
(TIF)

S6 Fig The tissue and developmental expression patterns of *OsARF5*, *OsARF11*, *OsARF12* and *OsARF16* by reverse transcriptase PCR. (A) The developmental expression patterns of *OsARF5*, *OsARF11*, *OsARF12* and *OsARF16* in NPB after sowing. (B) The tissue expression patterns of *OsARF5*, *OsARF11*, *OsARF12* and *OsARF16* in NPB. *Actin* was used as a loading control.
(TIF)

S7 Fig Identification of the *OsARF12* overexpression transgenic lines. (A) qRT-PCR results showing the *OsARF12* expression levels in the *OsARF12* OE lines. Three independent biological replicates were performed. The error bars indicate SD. #, number for the *OsARF12* OE line. (B) Western blot analysis of the OsARF12 protein level in the *OsARF12* OE lines. Actin was used as a loading control for proteins. (C) Phenotypes of non-RDV infected WT (ZH11) and *OsARF12* OE lines. Photos were taken at 4 weeks, Scale bars, 10 cm.
(TIF)

S8 Fig *osarf12* KO mutant is more susceptible to RDV infection. (A) Identification of *osarf12* KO rice plants. Genomic DNA sequencing of three *osarf12* KO lines. The mutations, ‘TCGAT’ deletion in line *osarf12* KO#1, ‘A’ insertion in line *osarf12* KO#5, ‘G’ insertion in line *osarf12* KO#6 which lead to premature termination of OsARF12. (B) Phenotypes of RDV-infected WT (ZH11) and *osarf12* KO lines. Photos were taken at 4 weeks after RDV-inoculation. The areas of white specks on the leaves represent the degree of disease symptoms. Scale bars, 10 cm (upper panel) and 1 cm (lower panel). (C) Schematic representation of plant height for the plants in (B). The average (±SD) values were obtained from three biological repeats. Different letters indicate significant difference (p< 0.05) based on the Tukey-Kramer HSD test. (D) RDV infection rates of the corresponding lines from one wpi to eight wpi. Inoculation assays were repeated three times, respectively. The error bars indicate SD. (E)Accumulation of RDV RNAs in the corresponding lines. The average (±SD) values were obtained from three biological repeats. The error bars indicate SD. (F) Accumulation of RDV proteins in the corresponding lines. Actin was used as a loading control for proteins.
(TIF)

S9 Fig GO biological processes enriched in differentially expressed genes of *OsIAA10*-related rice plants, *OsIAA10* RNAi #1 rice line and *OsIAA10P116L*-overexpressing M7 rice line. (A) Gene Ontology (GO) biological processes enriched in genes that are up-regulated in the *OsIAA10* RNAi #1 transgenic rice line. (B) Gene Ontology (GO) biological processes over-represented in genes that are down-regulated expression in the *OsIAA10P116L*-overexpressing M7 rice line. A homology-based annotation was performed by Blast2Go software. Briefly, all the gene sequences of the differentially expressed genes were blasted against the Swiss-Prot database with high E-value (1 × 10^−5^) and GO annotation was performed against the Gene Ontology Database. Fisher’s Exact Test was used to detect GO biological processes over-represented in the differentially expressed genes by using all identified genes as the background set. p.adjust <0.05.
(TIF)

S10 Fig SA signaling pathway related gene expression in these indicated rice plants. Expression of SA signaling pathway related genes in the *osiaa10* KO rice lines (A), *osarf5* mutant (B), *OsARF12* OE lines (C), *osarf12* mutant (D), *osarf16* mutant (E) and *osarf11* mutant (F).
(TIF)

S11 Fig Active oxygen species (ROS) accumulation level in these rice lines. Active oxygen species (ROS) accumulation level in *osiaa10* KO lines (A), *osarf12* and *OsARF12* OE lines (B).
(TIF)

S12 Fig The expression level of *OsWRKY13* in *osiaa10* KO and some *osarf* mutants. The expression level of *OsWRKY13* in *osiaa10* KO and *osarf12* KO mutants (A) and *osarf5*, *osarf11* and *osarf16* mutants (B).
(TIF)

S13 Fig Identification of the *oswrky13* KO transgenic lines. (A) Genomic DNA sequences of the three os*wrky13* KO lines. The mutations are ‘GTCGTCGCCC’ insertion in the line os*wrky13* KO#1, ‘C’ substitute to ‘A’ in the line os*wrky13* KO#2, ‘ACG’ and ‘GA’ deletion in the line os*wrky13* KO#18, respectively. All mutations cause premature termination of OsWRKY13. (B) Phenotypes of non-RDV infected WT (ZH11) and *oswrky13* KO lines. Photos were taken at 4 weeks, Scale bars, 10 cm.
(TIF)

S14 Fig A proposed model for the OsIAA10-OsARF12-OsWRKY13 module-mediated resistance against RDV. In healthy (uninfected) rice plants, when auxin concentration is low, OsARF12 is blocked by OsIAA10, then the expression of *OsWRKY13* can’t be activated. In RDV infected plants, although the higher level of free IAA content, but the viral P2 protein interacts with OsIAA10, blocking its association with OsTIR1, thus stabilizing OsIAA10 and preventing activation of *OsWRKY13* by OsARF12.
(TIF)

S1 Table Non-preference test for WT rice plants with indicated treatment. (DOCX)

S2 Table Record of the number of rice plants showing RDV symptoms at time course. (DOCX)

S3 Table Constructs list. (DOCX)

S4 Table Primers list. (DOCX)
