# Supplementary figures and images for: Auxin response factors (ARFs) differentially regulate rice antiviral immune response against rice dwarf virus
Source: PLoS Pathog. 2020 Dec 2;16(12):e1009118. doi: 10.1371/journal.ppat.1009118 (PMC7735678; doi:10.1371/journal.ppat.1009118)

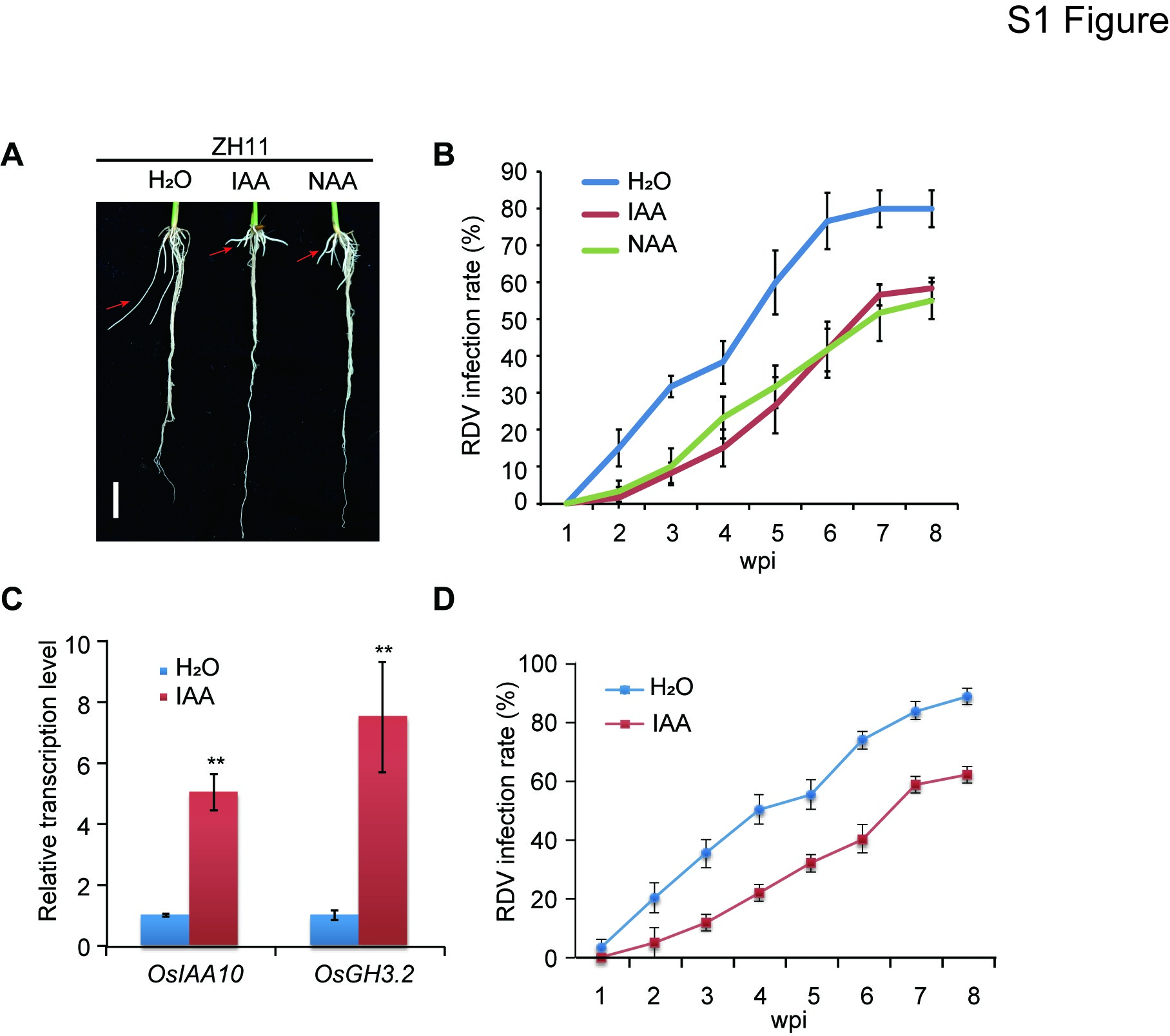

Supplement: S1 Fig — (A) Elongation of crown roots is inhibited by treatment with IAA or NAA in ZH11. Photograph was taken with a Nicon camera. Scale bar, 2.5 cm; Red arrows indicate the newly grown crown roots. (B) RDV infection rates in ZH11 rice plants pretreated with auxin. Time course of RDV infection rates in rice plants pretreated with H2O, IAA or NAA from one to eight wpi. Inoculation assays were repeated three times. The error bars indicate SD. (C) qRT-PCR assay showing the relative expression levels of OsIAA10 and OsGH3.2 after pre-spraying with water or IAA in ZH11. OsEF1a was used as a reference. The average (± SD) values were obtained from three biological repeats. Significant differences (*P< 0.05, **P< 0.01) are indicated based on Students’ t-test. (D) Time course of RDV infection rates for ZH11 rice plants pre-spraying with water or IAA from one to eight wpi. Inoculation assays were repeated three times. The error bars indicate SD. (TIF) [file ppat.1009118.s001.tif]

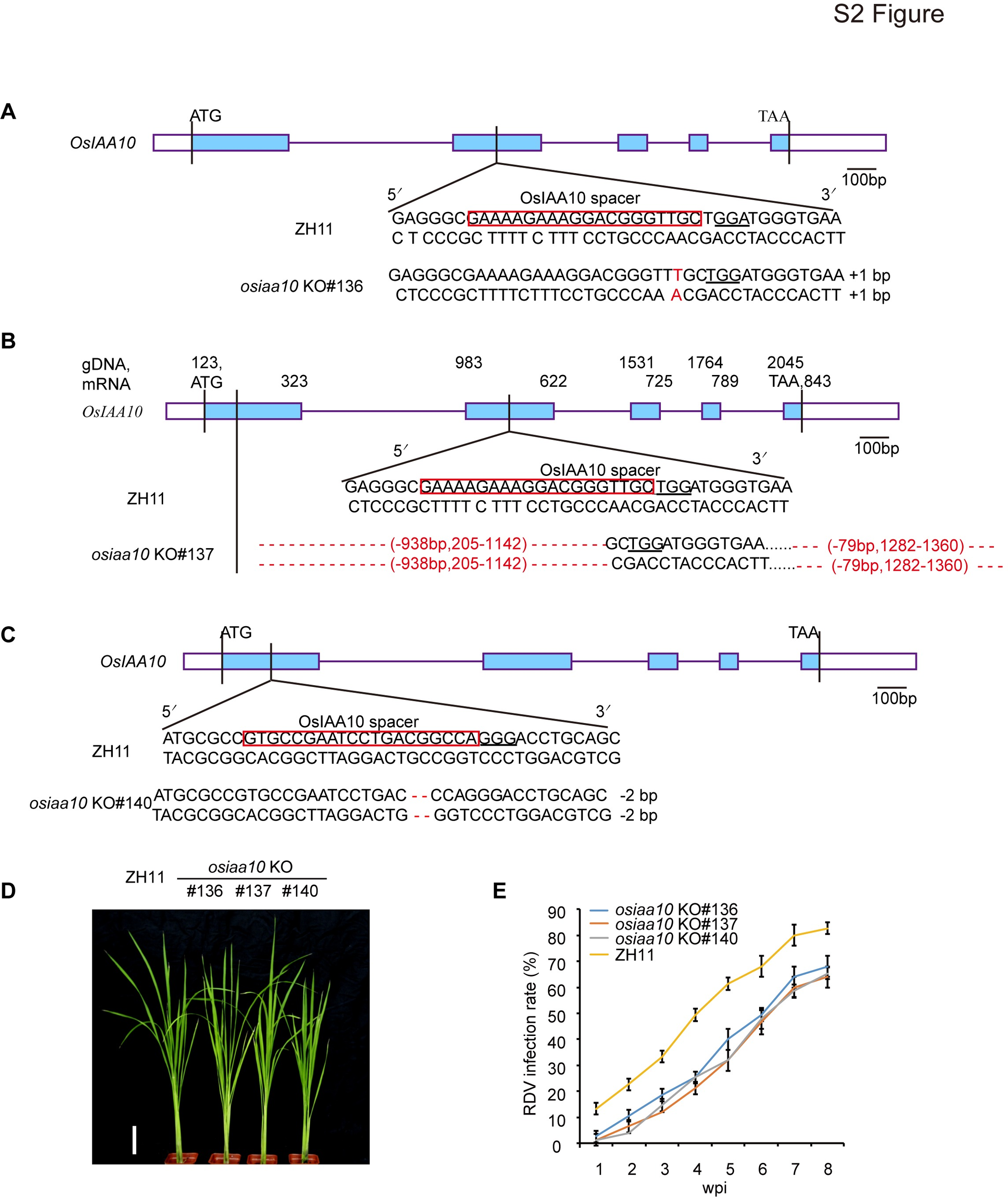

Supplement: S2 Fig — (A) ‘T’ insertion in the line osiaa10 KO #136. The mutation leads to premature termination of OsIAA10. (B) 938 bp and 79 bp deletion in the line osiaa10 KO #137, causing premature termination of OsIAA10. (C) ‘GG’ deletion in the line osiaa10 KO #140, causing premature termination of OsIAA10. (D) Phenotypes of non-RDV infected WT (ZH11) and osiaa10 KO lines. Photos were taken at 4 weeks, Scale bars, 10 cm. (E) RDV infection rates in the osiaa10 KO rice plants. Time course of RDV infection rates in the osiaa10 KO rice plants from one to eight wpi. Inoculation assays were repeated three times. The error bars indicate SD. (TIF) [file ppat.1009118.s002.tif]

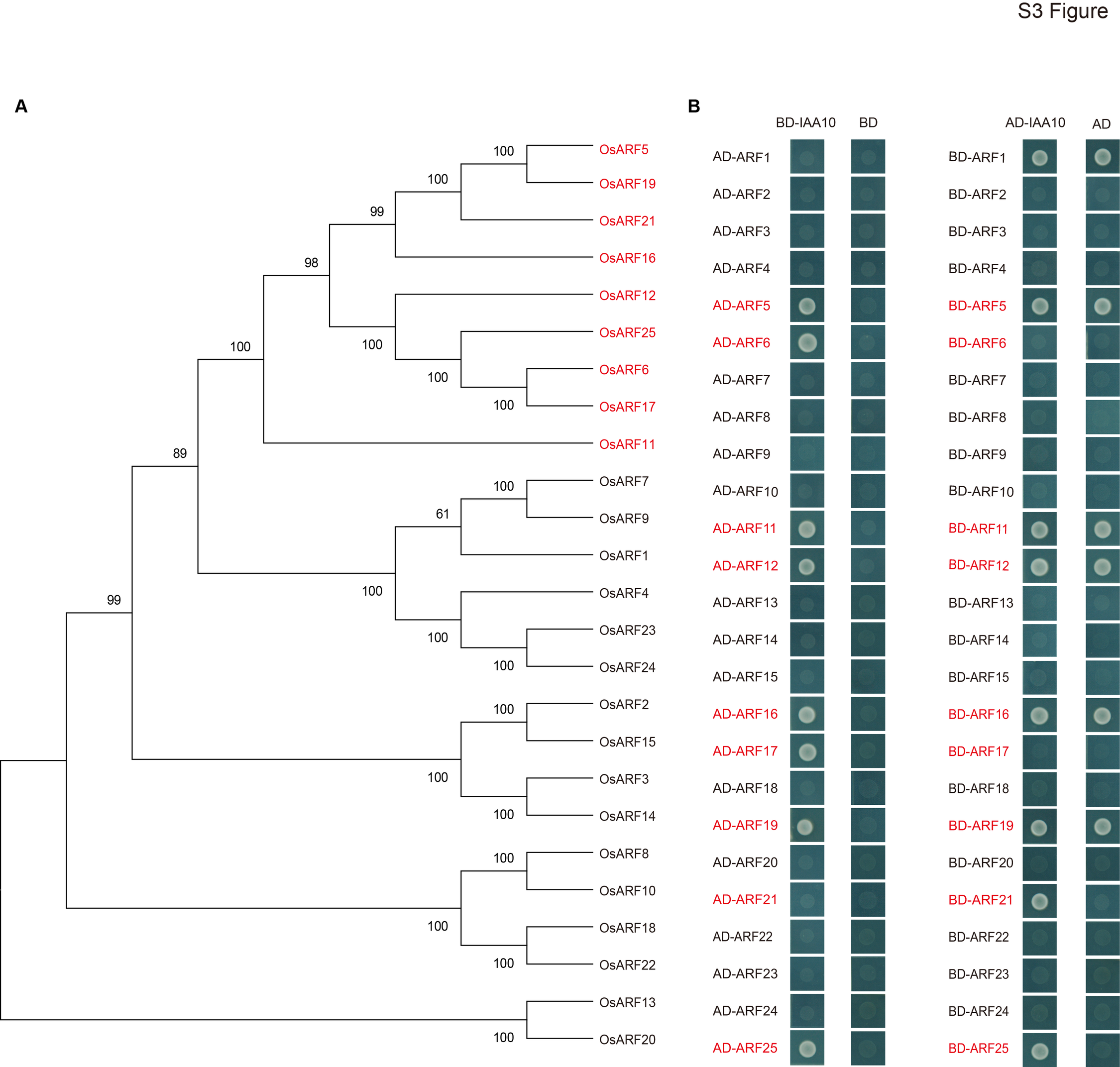

Supplement: S3 Fig — (A) The phylogenetic tree of OsARFs. Phylogenetic relationship among the rice OsARF proteins. The unrooted tree was generated using ClustalX program by neighbor-joining method. Bootstrap values form 100 replicates are indicated at each node. (B) Y2H screen for OsARFs that interact with OsIAA10. Yeast two-hybrid assay for confirming the OsARF and OsIAA10 interaction. The bait protein OsARF is expressed as a GAL4 DNA binding domain fusion, and the OsIAA10 is expressed as GAL4 DNA activation domain fusions in yeast AH109 cells. Positive interaction is indicated by the ability of cells to grow on medium lacking His (-H) and Adeline (-Ade). Vectors expressing the GAL4 binding domain (BD) or GAL4 activating domain (AD) are used as negative controls. SD, synthetic dropout medium; -L, lacking Leu; -W, lacking Trp. (TIF) [file ppat.1009118.s003.tif]

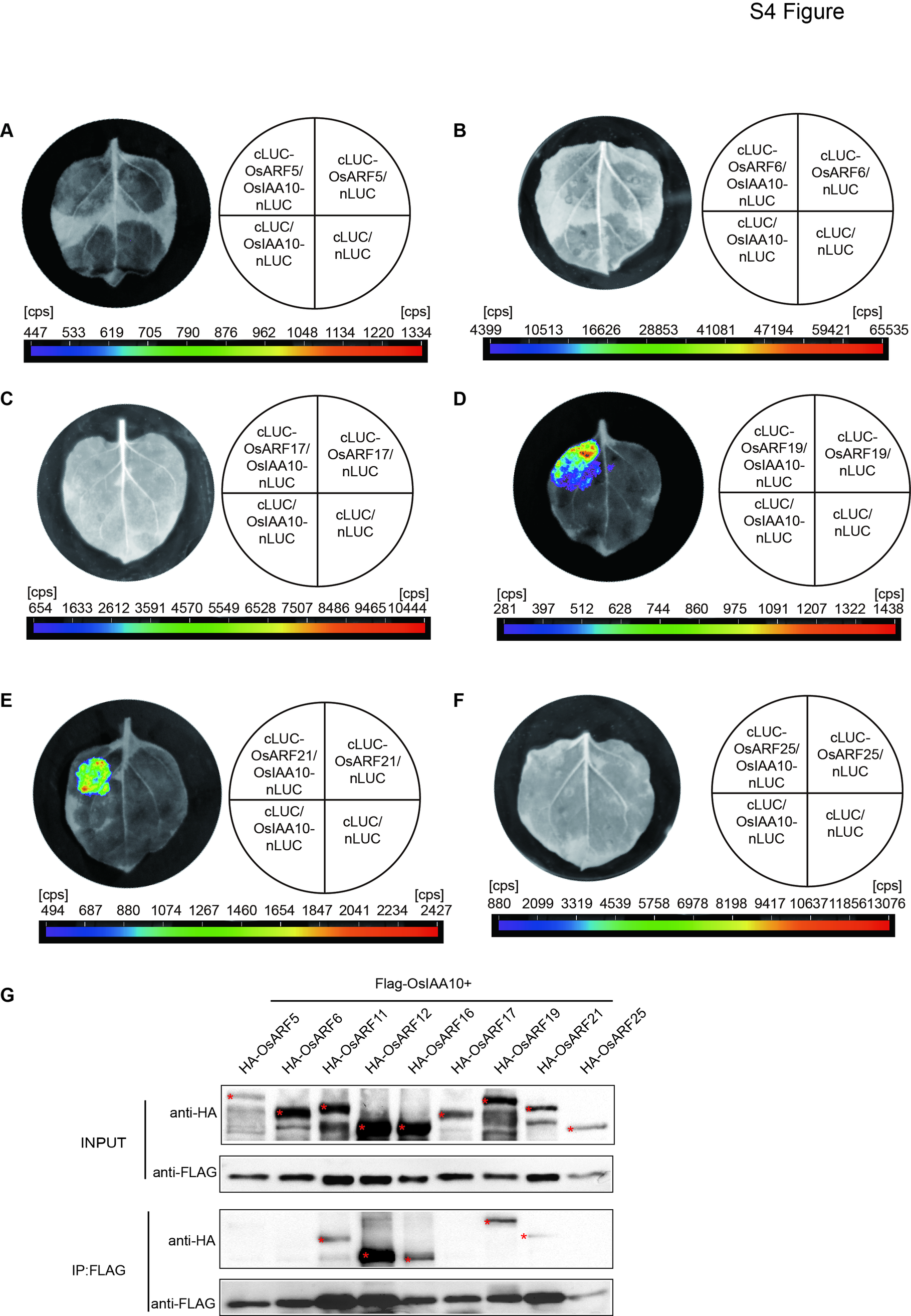

Supplement: S4 Fig — (A)There is no interaction between OsIAA10 and OsARF5. (B) There is no interaction between OsIAA10 and OsARF6. (C) There is no interaction between OsIAA10 and OsARF17. (D) OsIAA10 interacts with OsARF19 in plants. (E) OsIAA10 interacts with OsARF21 in plants. (F) There is no interaction between OsIAA10 and OsARF25. The left diagram indicates the leaf panels that were infiltrated with A. tumefaciens containing the different combinations of indicated constructs. Cps indicates signal counts per second. (G) Co-immunoprecipitation confirmed the interaction between OsIAA10 and OsARF11, OsARF12, OsARF16, OsARF19, OsARF21. (TIF) [file ppat.1009118.s004.tif]

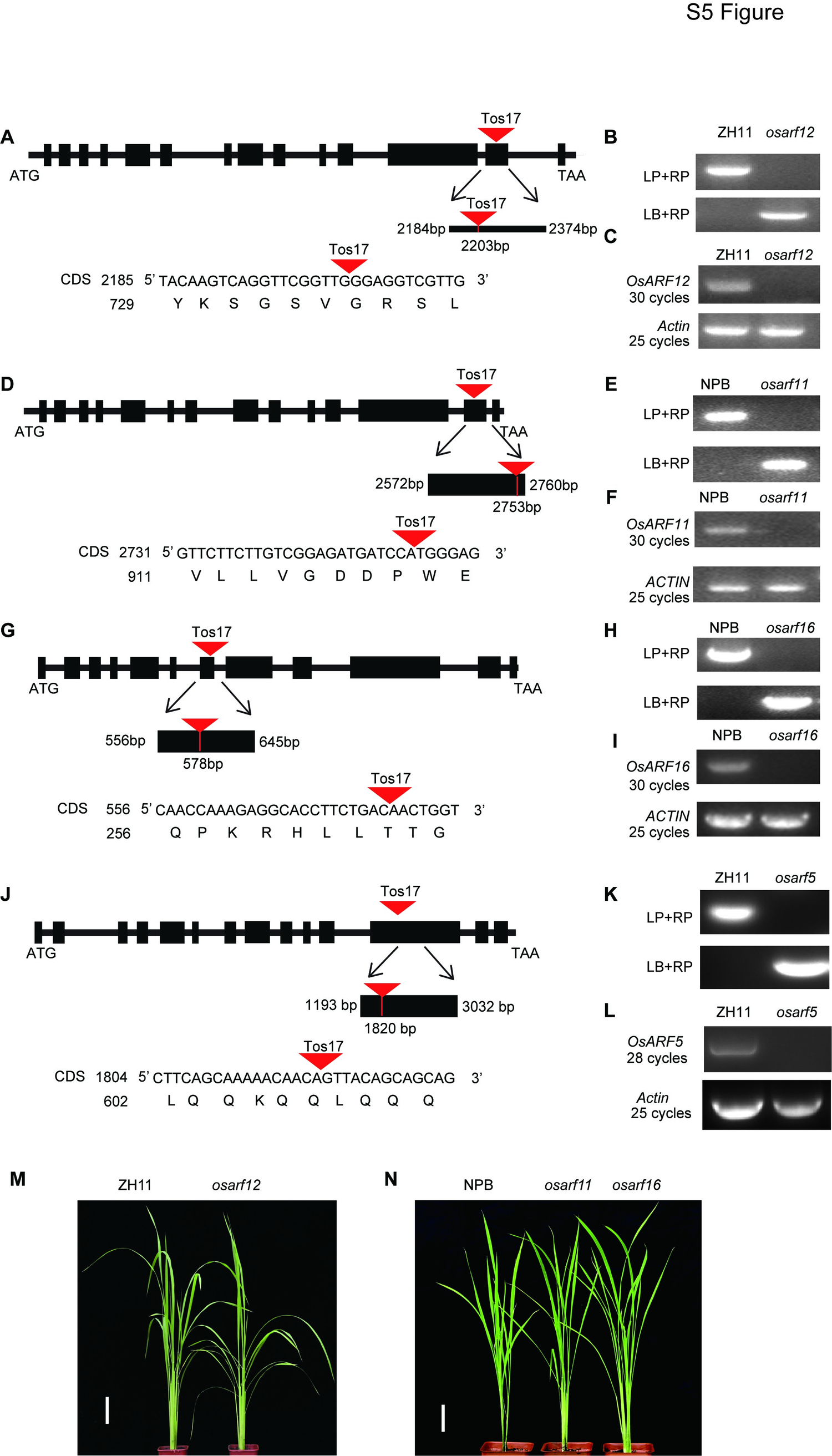

Supplement: S5 Fig — (A) Tos17 insertion site in the osarf12 mutant. The black box represents the exon, and the black line represents the intron. (B) PCR analysis confirms the integration of Tos17 in OsARF12. The lower bands indicate that Tos17 is inserted into the OsARF12 genic region in the osarf12 mutant. (C) Semi-quantitative RT-PCR analysis shows that the OsARF12 gene is not expressed in the mutant. Actin was used as a loading control. (D) Tos17 insertion site in the osarf11 mutant. The black box represents the exon, and the black line represents the intron. (E) PCR analysis confirms the integration of Tos17 in OsARF11. The lower bands indicate that Tos17 is inserted into the OsARF11 genic region in the osarf11 mutant. (F) RT-PCR analysis showing that the OsARF11 gene is not expressed in the mutant. Actin was used as a loading control. (G) Tos17 insertion site in the osarf16 mutant. The black box represents the exon, and the black line represents the intron. (H) PCR analysis confirms the integration of Tos17 in OsARF16. The lower bands indicate that Tos17 is inserted into the OsARF16 genomic region in the osarf16 mutant. (I) Semi-quantitative RT-PCR analysis shows that the OsARF16 gene is not expressed in the mutant. Actin was used as a loading control. (J) Tos17 insertion site in the osarf5 mutant. The black box represents the exon, and the black line represents the intron. (K) PCR analysis confirms the integration of Tos17 in OsARF5. The lower bands indicate that Tos17 is inserted into the OsARF5 genic region in the osarf11 mutant. (L) Semi-quantitative RT-PCR analysis shows that the OsARF5 gene is not expressed in the mutant. Actin was used as a loading control. (M) Phenotypes of non-RDV infected WT (ZH11) and osarf12 mutant lines. Photos were taken at 4 weeks, Scale bars, 10 cm. (N) Phenotypes of non-RDV infected WT (NPB) and osarf11, osarf16 mutant lines. Photos were taken at 4 weeks, Scale bars, 10 cm. (TIF) [file ppat.1009118.s005.tif]

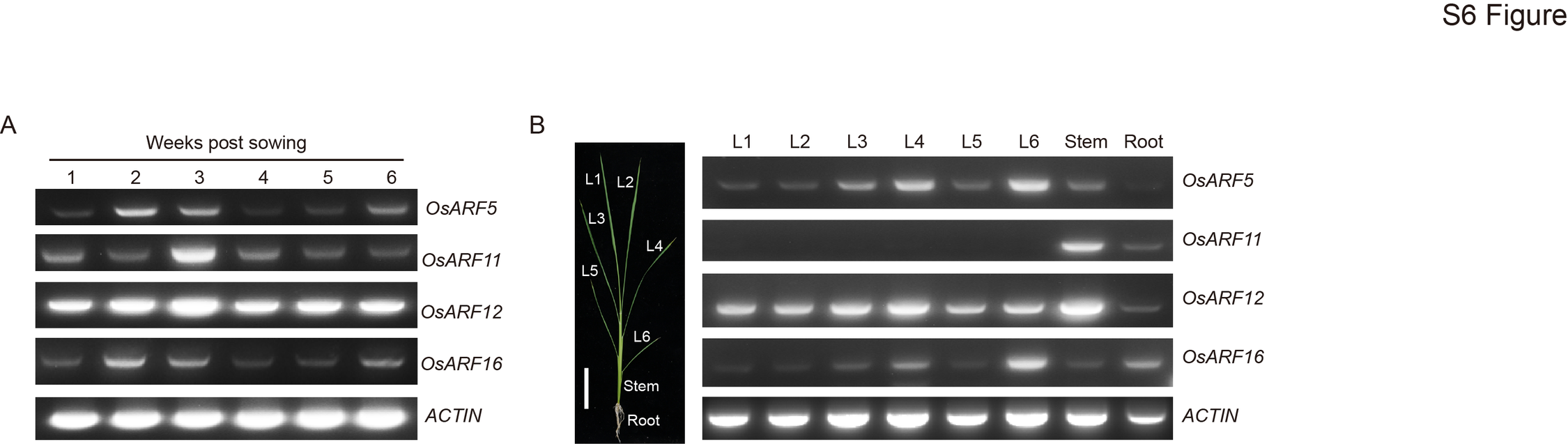

Supplement: S6 Fig — (A) The developmental expression patterns of OsARF5, OsARF11, OsARF12 and OsARF16 in NPB after sowing. (B) The tissue expression patterns of OsARF5, OsARF11, OsARF12 and OsARF16 in NPB. Actin was used as a loading control. (TIF) [file ppat.1009118.s006.tif]

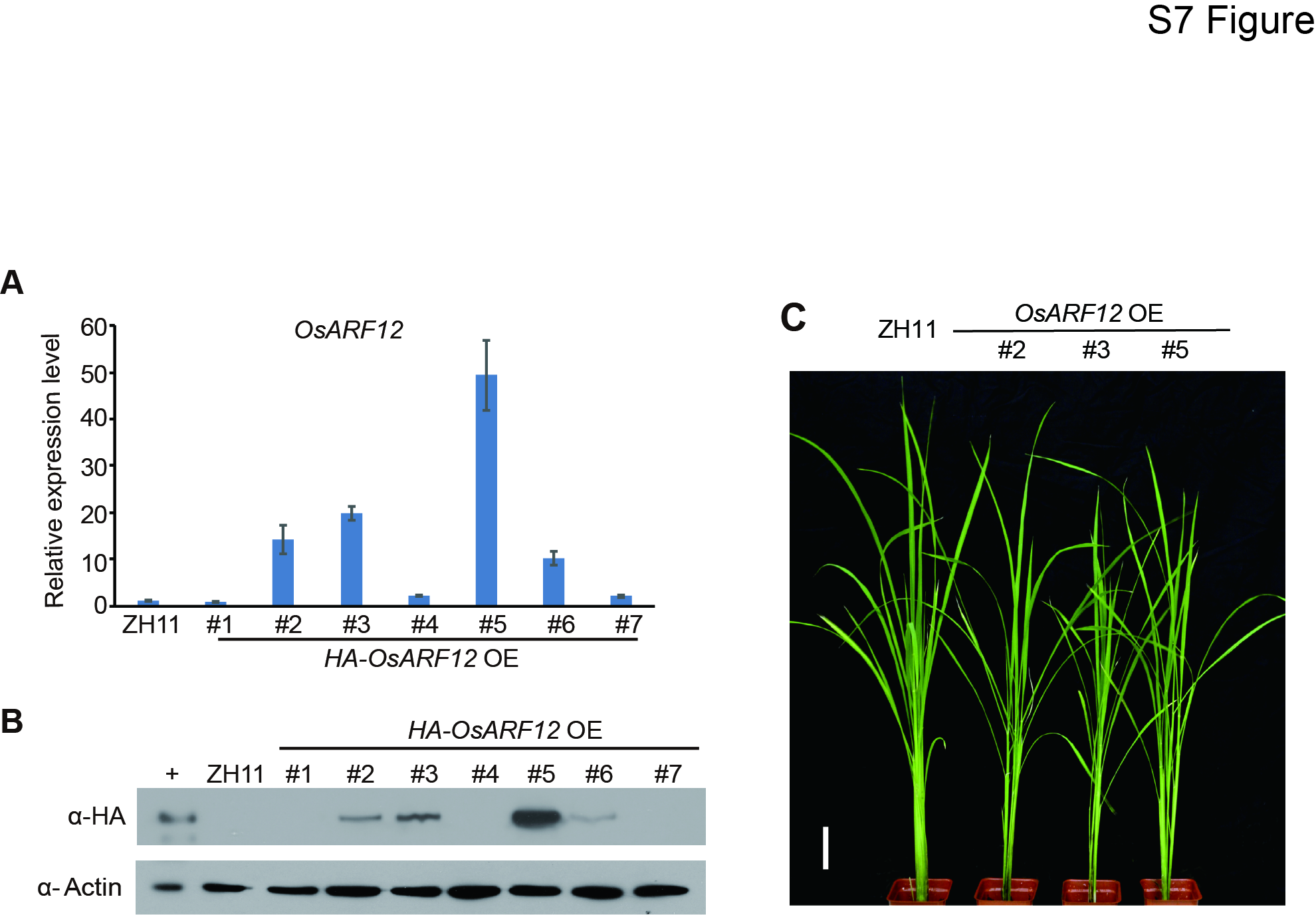

Supplement: S7 Fig — (A) qRT-PCR results showing the OsARF12 expression levels in the OsARF12 OE lines. Three independent biological replicates were performed. The error bars indicate SD. #, number for the OsARF12 OE line. (B) Western blot analysis of the OsARF12 protein level in the OsARF12 OE lines. Actin was used as a loading control for proteins. (C) Phenotypes of non-RDV infected WT (ZH11) and OsARF12 OE lines. Photos were taken at 4 weeks, Scale bars, 10 cm. (TIF) [file ppat.1009118.s007.tif]

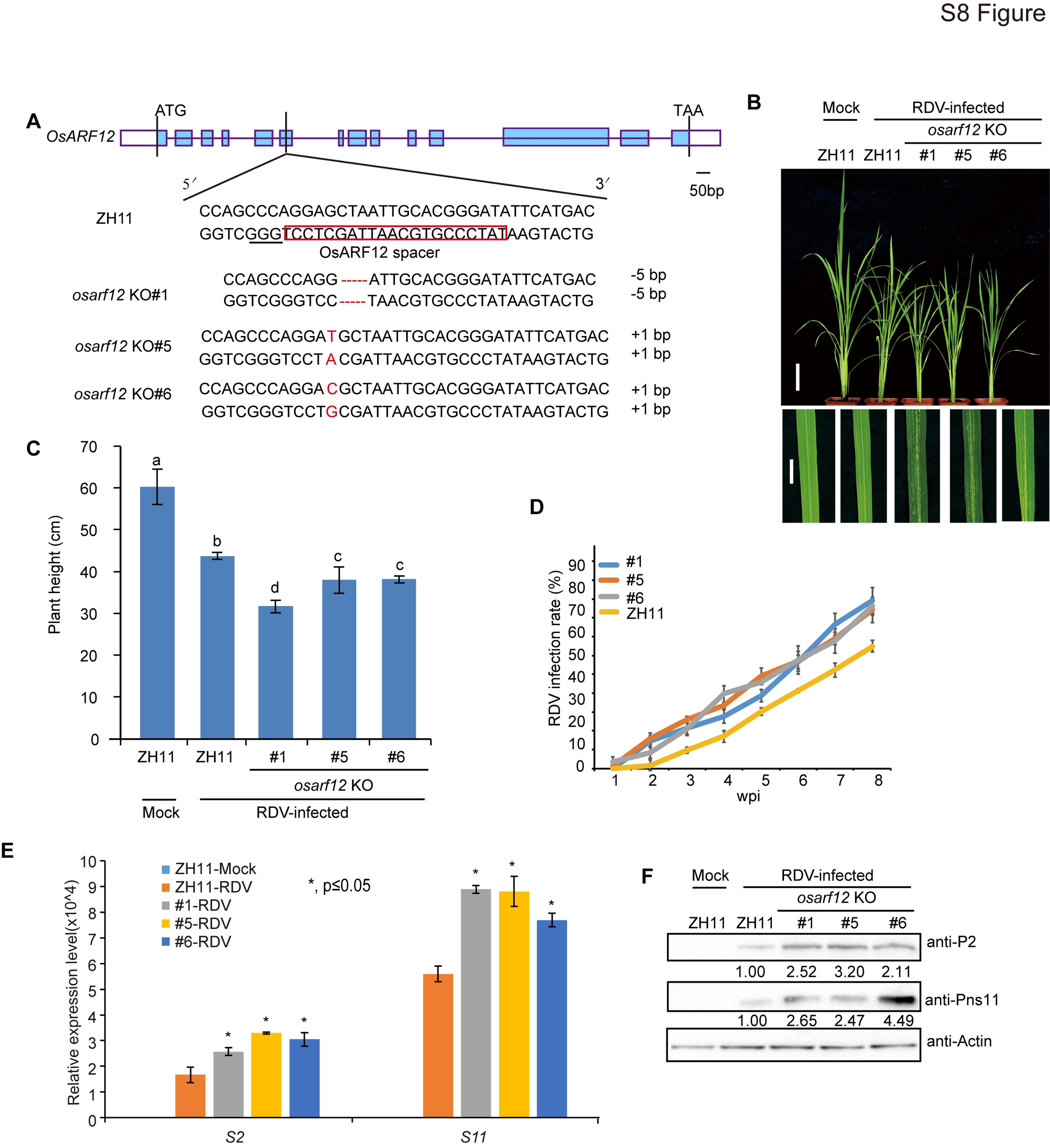

Supplement: S8 Fig — (A) Identification of osarf12 KO rice plants. Genomic DNA sequencing of three osarf12 KO lines. The mutations, ‘TCGAT’ deletion in line osarf12 KO#1, ‘A’ insertion in line osarf12 KO#5, ‘G’ insertion in line osarf12 KO#6 which lead to premature termination of OsARF12. (B) Phenotypes of RDV-infected WT (ZH11) and osarf12 KO lines. Photos were taken at 4 weeks after RDV-inoculation. The areas of white specks on the leaves represent the degree of disease symptoms. Scale bars, 10 cm (upper panel) and 1 cm (lower panel). (C) Schematic representation of plant height for the plants in (B). The average (±SD) values were obtained from three biological repeats. Different letters indicate significant difference (p< 0.05) based on the Tukey-Kramer HSD test. (D) RDV infection rates of the corresponding lines from one wpi to eight wpi. Inoculation assays were repeated three times, respectively. The error bars indicate SD. (E)Accumulation of RDV RNAs in the corresponding lines. The average (±SD) values were obtained from three biological repeats. The error bars indicate SD. (F) Accumulation of RDV proteins in the corresponding lines. Actin was used as a loading control for proteins. (TIF) [file ppat.1009118.s008.tif]

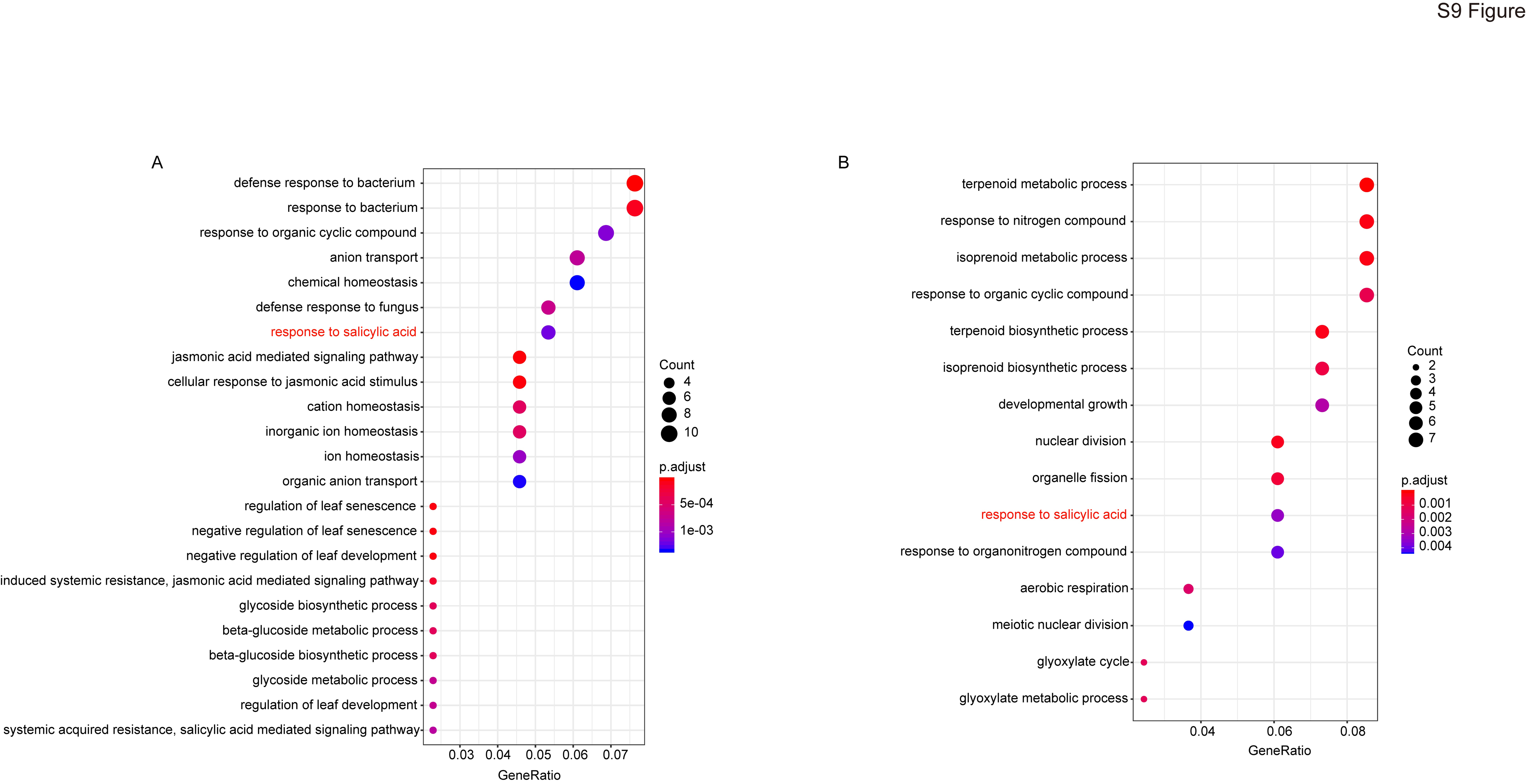

Supplement: S9 Fig — (A) Gene Ontology (GO) biological processes enriched in genes that are up-regulated in the OsIAA10 RNAi #1 transgenic rice line. (B) Gene Ontology (GO) biological processes over-represented in genes that are down-regulated expression in the OsIAA10P116L-overexpressing M7 rice line. A homology-based annotation was performed by Blast2Go software. Briefly, all the gene sequences of the differentially expressed genes were blasted against the Swiss-Prot database with high E-value (1 × 10^−5^) and GO annotation was performed against the Gene Ontology Database. Fisher’s Exact Test was used to detect GO biological processes over-represented in the differentially expressed genes by using all identified genes as the background set. p.adjust <0.05. (TIF) [file ppat.1009118.s009.tif]

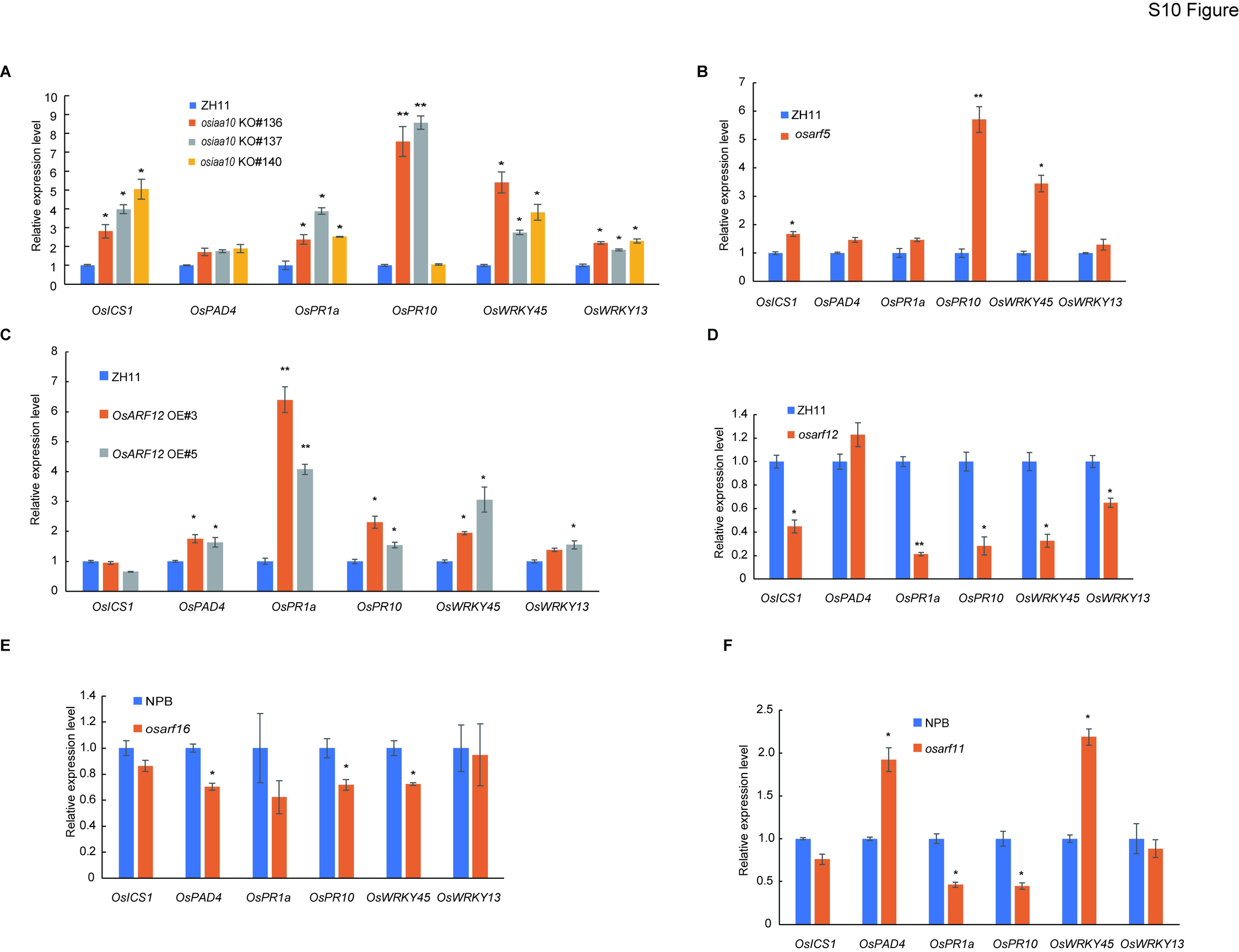

Supplement: S10 Fig — Expression of SA signaling pathway related genes in the osiaa10 KO rice lines (A), osarf5 mutant (B), OsARF12 OE lines (C), osarf12 mutant (D), osarf16 mutant (E) and osarf11 mutant (F). (TIF) [file ppat.1009118.s010.tif]

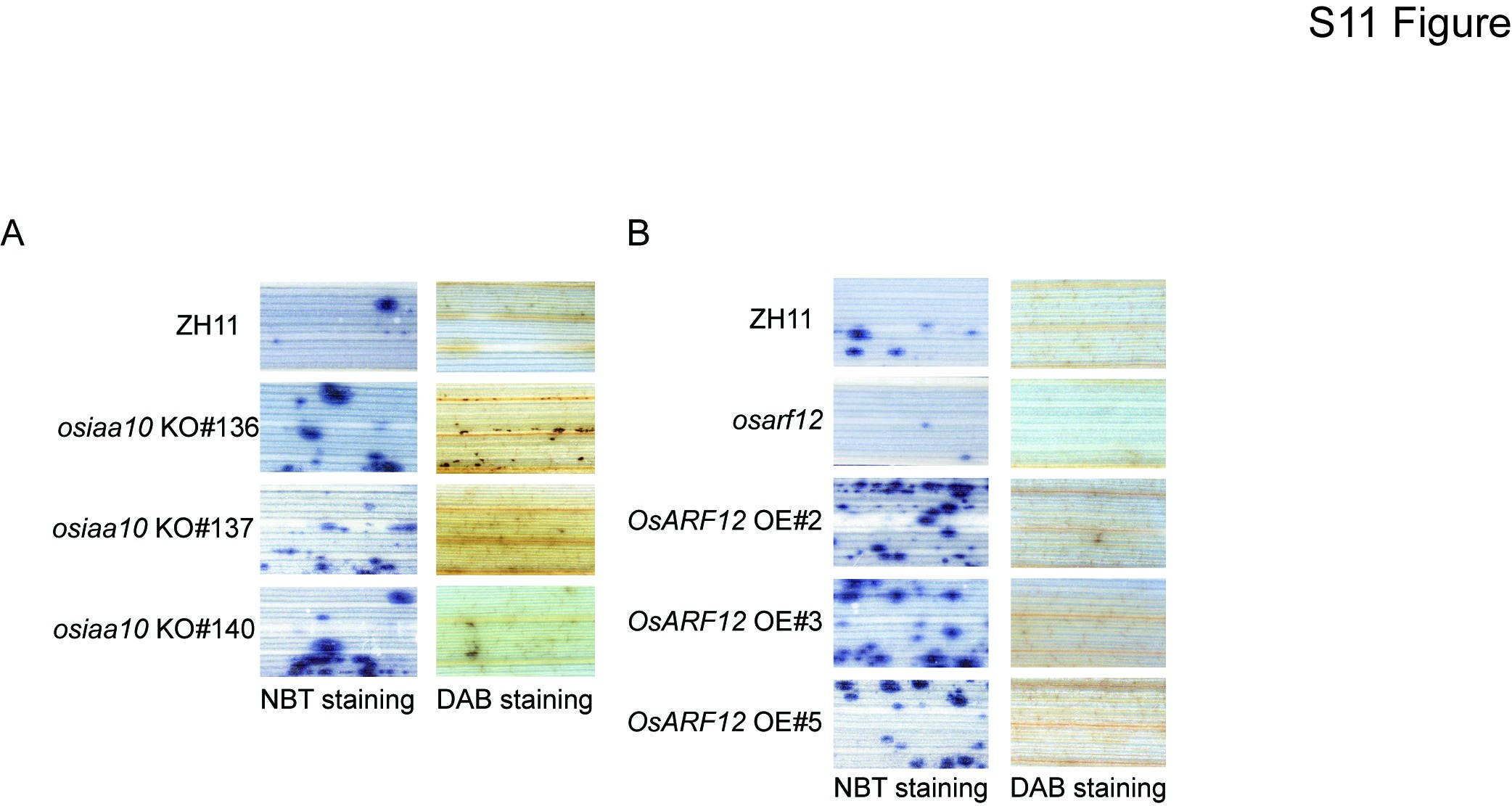

Supplement: S11 Fig — Active oxygen species (ROS) accumulation level in osiaa10 KO lines (A), osarf12 and OsARF12 OE lines (B). (TIF) [file ppat.1009118.s011.tif]

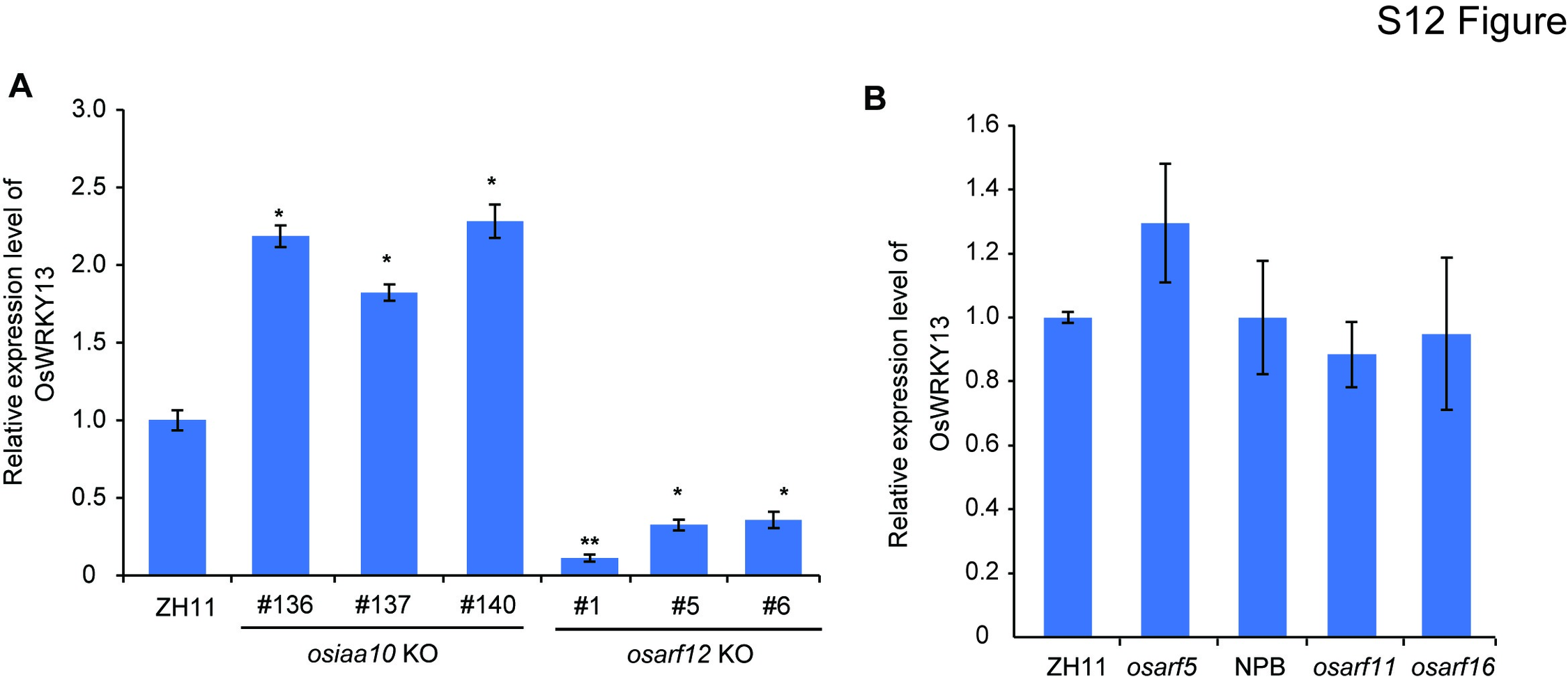

Supplement: S12 Fig — The expression level of OsWRKY13 in osiaa10 KO and osarf12 KO mutants (A) and osarf5, osarf11 and osarf16 mutants (B). (TIF) [file ppat.1009118.s012.tif]

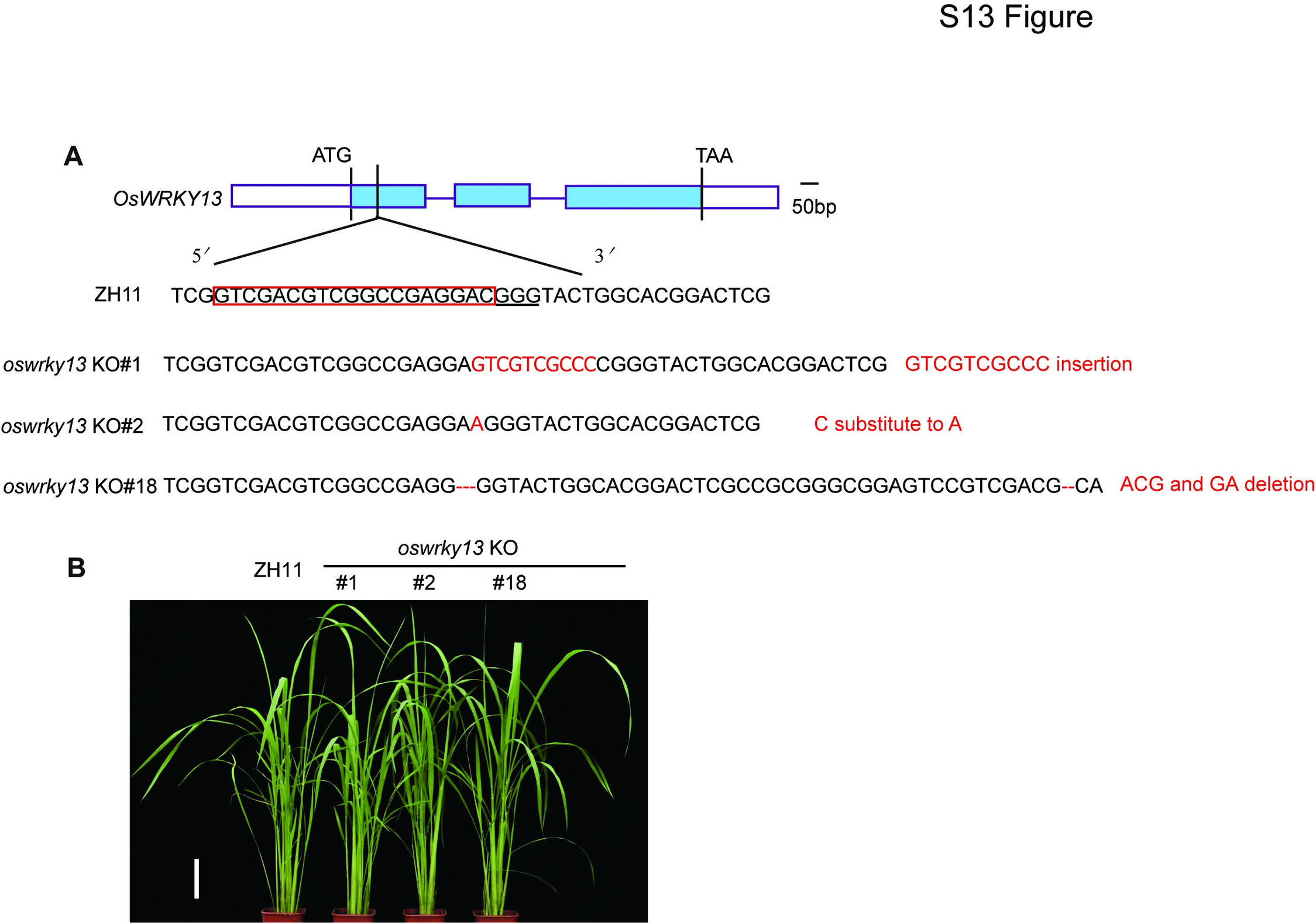

Supplement: S13 Fig — (A) Genomic DNA sequences of the three oswrky13 KO lines. The mutations are ‘GTCGTCGCCC’ insertion in the line oswrky13 KO#1, ‘C’ substitute to ‘A’ in the line oswrky13 KO#2, ‘ACG’ and ‘GA’ deletion in the line oswrky13 KO#18, respectively. All mutations cause premature termination of OsWRKY13. (B) Phenotypes of non-RDV infected WT (ZH11) and oswrky13 KO lines. Photos were taken at 4 weeks, Scale bars, 10 cm. (TIF) [file ppat.1009118.s013.tif]

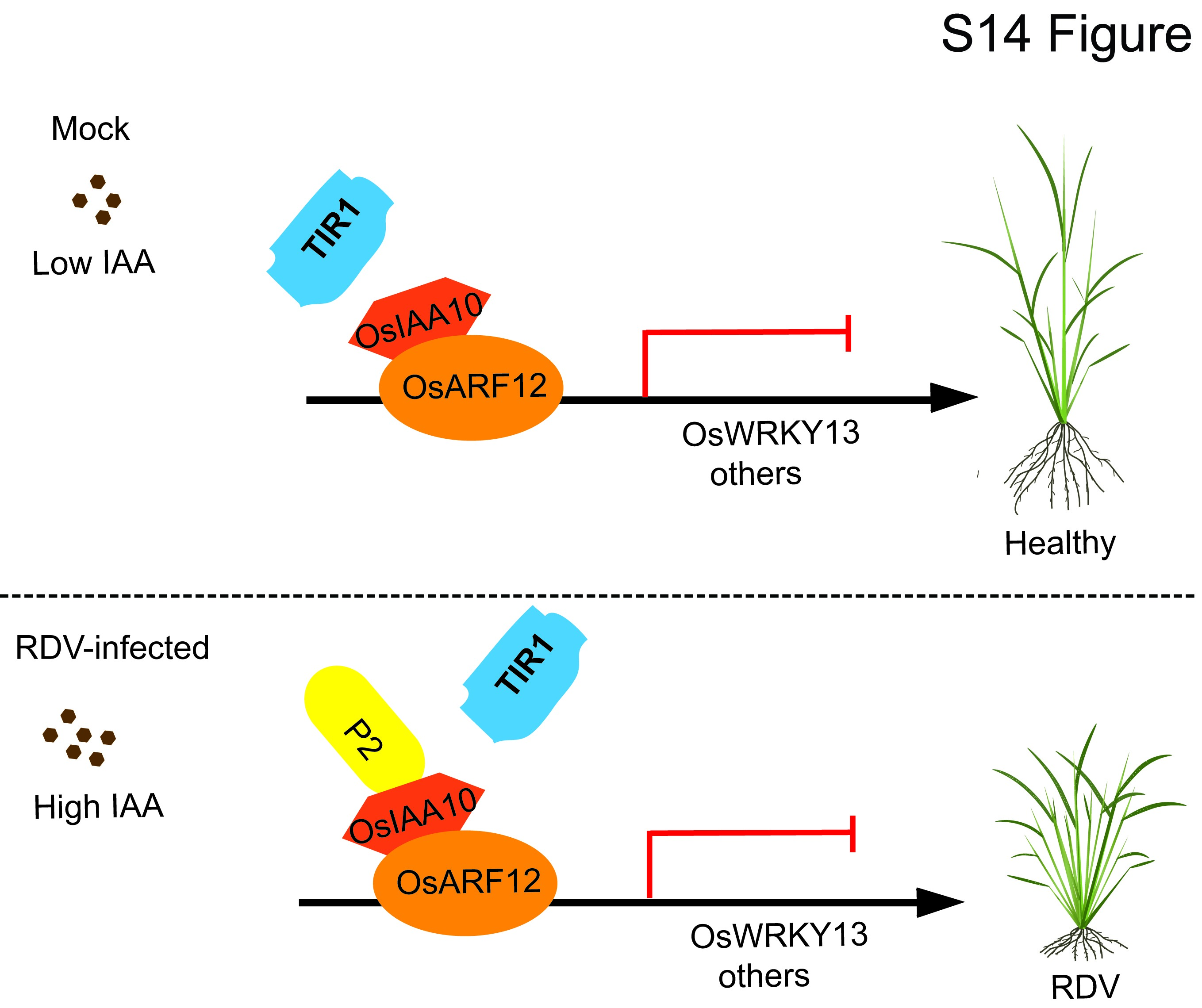

Supplement: S14 Fig — In healthy (uninfected) rice plants, when auxin concentration is low, OsARF12 is blocked by OsIAA10, then the expression of OsWRKY13 can’t be activated. In RDV infected plants, although the higher level of free IAA content, but the viral P2 protein interacts with OsIAA10, blocking its association with OsTIR1, thus stabilizing OsIAA10 and preventing activation of OsWRKY13 by OsARF12. (TIF) [file ppat.1009118.s014.tif]
