# Supplementary material for: Auxin response factors (ARFs) differentially regulate rice antiviral immune response against rice dwarf virus
Source: PLoS Pathog. 2020 Dec 2;16(12):e1009118. doi: 10.1371/journal.ppat.1009118 (PMC7735678; doi:10.1371/journal.ppat.1009118)
Supplement: S1 Table — (DOCX) [file ppat.1009118.s015.docx]

**Supplemental** **Table 1. Non-preference test for WT rice plants with indicated treatment.**

| Varieties | Non-preference |
| --- | --- |
| H_2_O | 2.10^a^ |
| IAA | 1.85^a^ |
| NAA | 2.05^a^ |

*1, Non-preference was indicated by the number of leafhoppers settled on the individual plant.

*2, “a” means there is no significant difference (P value> 0.05) between these data.
