# Supplementary material for: Auxin response factors (ARFs) differentially regulate rice antiviral immune response against rice dwarf virus
Source: PLoS Pathog. 2020 Dec 2;16(12):e1009118. doi: 10.1371/journal.ppat.1009118 (PMC7735678; doi:10.1371/journal.ppat.1009118)
Supplement: S2 Table — (DOCX) [file ppat.1009118.s016.docx]

**S2 Table. Record of the number of rice plants showing RDV symptoms at time course.**

| Time | Repeat^#^ | H_2_O | IAA | NAA |
| --- | --- | --- | --- | --- |
| 1 wpi* | R1 | 0 | 0 | 0 |
|  | R2 | 0 | 0 | 0 |
|  | R3 | 0 | 0 | 0 |
| 2 wpi | R1 | 4 | 1 | 1 |
|  | R2 | 3 | 0 | 0 |
|  | R3 | 2 | 0 | 1 |
| 3 wpi | R1 | 6 | 2 | 3 |
|  | R2 | 7 | 1 | 1 |
|  | R3 | 6 | 2 | 2 |
| 4 wpi | R1 | 9 | 4 | 6 |
|  | R2 | 7 | 2 | 4 |
|  | R3 | 7 | 3 | 4 |
| 5 wpi | R1 | 13 | 7 | 7 |
|  | R2 | 10 | 5 | 7 |
|  | R3 | 13 | 4 | 5 |
| 6 wpi | R1 | 15 | 10 | 9 |
|  | R2 | 14 | 8 | 9 |
|  | R3 | 17 | 7 | 7 |
| 7 wpi | R1 | 16 | 11 | 10 |
|  | R2 | 15 | 12 | 12 |
|  | R3 | 17 | 11 | 9 |
| 8 wpi | R1 | 16 | 12 | 11 |
|  | R2 | 15 | 12 | 12 |
|  | R3 | 17 | 11 | 10 |

^#^ For each repeat, 20 seedlings were inoculated with viruliferous leafhopper.

* wpi, week-post-inoculation.

| Time | Repeat^#^ | H_2_O | IAA |
| --- | --- | --- | --- |
| 1 wpi | R1 | 1 | 0 |
|  | R2 | 1 | 0 |
|  | R3 | 0 | 0 |
| 2 wpi | R1 | 4 | 1 |
|  | R2 | 5 | 2 |
|  | R3 | 3 | 0 |
| 3 wpi | R1 | 8 | 2 |
|  | R2 | 7 | 3 |
|  | R3 | 6 | 2 |
| 4 wpi | R1 | 9 | 4 |
|  | R2 | 11 | 5 |
|  | R3 | 10 | 4 |
| 5 wpi | R1 | 10 | 6 |
|  | R2 | 12 | 7 |
|  | R3 | 11 | 6 |
| 6 wpi | R1 | 15 | 8 |
|  | R2 | 14 | 9 |
|  | R3 | 15 | 7 |
| 7 wpi | R1 | 17 | 12 |
|  | R2 | 16 | 12 |
|  | R3 | 17 | 11 |
| 8 wpi | R1 | 18 | 13 |
|  | R2 | 18 | 12 |
|  | R3 | 17 | 12 |

^#^ For each repeat, 25 seedlings were inoculated with viruliferous leafhopper.

| Time | Repeat^#^ | *osiaa10* KO#136 | *osiaa10* KO#137 | *osiaa10* KO#140 | WT(ZH11) |
| --- | --- | --- | --- | --- | --- |
| 1 wpi | R1 | 1 | 1 | 0 | 3 |
|  | R2 | 0 | 0 | 1 | 3 |
|  | R3 | 1 | 0 | 0 | 4 |
| 2 wpi | R1 | 3 | 2 | 1 | 6 |
|  | R2 | 3 | 1 | 1 | 5 |
|  | R3 | 2 | 2 | 1 | 6 |
| 3 wpi | R1 | 5 | 3 | 3 | 8 |
|  | R2 | 4 | 3 | 4 | 8 |
|  | R3 | 5 | 3 | 4 | 9 |
| 4 wpi | R1 | 7 | 6 | 6 | 12 |
|  | R2 | 6 | 5 | 7 | 13 |
|  | R3 | 6 | 5 | 6 | 12 |
| 5 wpi | R1 | 11 | 7 | 7 | 16 |
|  | R2 | 9 | 8 | 9 | 15 |
|  | R3 | 10 | 9 | 8 | 15 |
| 6 wpi | R1 | 12 | 13 | 12 | 17 |
|  | R2 | 13 | 11 | 13 | 18 |
|  | R3 | 12 | 11 | 11 | 16 |
| 7 wpi | R1 | 17 | 16 | 15 | 20 |
|  | R2 | 15 | 15 | 15 | 21 |
|  | R3 | 16 | 14 | 14 | 19 |
| 8 wpi | R1 | 18 | 17 | 16 | 20 |
|  | R2 | 17 | 15 | 17 | 21 |
|  | R3 | 16 | 16 | 16 | 21 |

^#^ For each repeat, 25 seedlings were inoculated with viruliferous leafhopper.

| Time | Repeat^#^ | *osarf11* | *osarf16* | WT(NPB) |
| --- | --- | --- | --- | --- |
| 1 wpi | R1 | 0 | 0 | 0 |
|  | R2 | 0 | 0 | 0 |
|  | R3 | 0 | 0 | 0 |
| 2 wpi | R1 | 1 | 3 | 2 |
|  | R2 | 0 | 2 | 3 |
|  | R3 | 2 | 4 | 4 |
| 3 wpi | R1 | 2 | 9 | 5 |
|  | R2 | 3 | 8 | 6 |
|  | R3 | 3 | 10 | 7 |
| 4 wpi | R1 | 4 | 14 | 10 |
|  | R2 | 5 | 12 | 12 |
|  | R3 | 6 | 14 | 11 |
| 5 wpi | R1 | 7 | 16 | 13 |
|  | R2 | 7 | 15 | 13 |
|  | R3 | 8 | 16 | 13 |
| 6 wpi | R1 | 10 | 18 | 15 |
|  | R2 | 11 | 17 | 17 |
|  | R3 | 12 | 17 | 16 |
| 7 wpi | R1 | 15 | 20 | 17 |
|  | R2 | 14 | 21 | 18 |
|  | R3 | 16 | 19 | 19 |
| 8 wpi | R1 | 17 | 21 | 20 |
|  | R2 | 15 | 22 | 18 |
|  | R3 | 16 | 20 | 19 |

^#^ For each repeat, 25 seedlings were inoculated with viruliferous leafhopper.

| Time | Repeat^#^ | *OsARF12*  OE#2 | *OsARF12*  OE#3 | *OsARF12*  OE#5 | WT(ZH11) |
| --- | --- | --- | --- | --- | --- |
| 1 wpi | R1 | 0 | 0 | 1 | 3 |
|  | R2 | 0 | 1 | 1 | 2 |
|  | R3 | 1 | 0 | 0 | 3 |
| 2 wpi | R1 | 1 | 1 | 1 | 5 |
|  | R2 | 2 | 2 | 2 | 4 |
|  | R3 | 1 | 2 | 2 | 5 |
| 3 wpi | R1 | 2 | 2 | 2 | 6 |
|  | R2 | 3 | 2 | 2 | 5 |
|  | R3 | 3 | 2 | 2 | 5 |
| 4 wpi | R1 | 3 | 3 | 3 | 9 |
|  | R2 | 6 | 3 | 3 | 8 |
|  | R3 | 4 | 3 | 3 | 7 |
| 5 wpi | R1 | 5 | 7 | 4 | 13 |
|  | R2 | 7 | 6 | 5 | 11 |
|  | R3 | 5 | 9 | 5 | 12 |
| 6 wpi | R1 | 8 | 7 | 6 | 14 |
|  | R2 | 9 | 7 | 7 | 15 |
|  | R3 | 7 | 6 | 7 | 16 |
| 7 wpi | R1 | 12 | 11 | 7 | 16 |
|  | R2 | 10 | 8 | 8 | 17 |
|  | R3 | 9 | 8 | 8 | 19 |
| 8 wpi | R1 | 14 | 12 | 9 | 19 |
|  | R2 | 15 | 13 | 13 | 20 |
|  | R3 | 13 | 13 | 13 | 21 |

^#^ For each repeat, 25 seedlings were inoculated with viruliferous leafhopper.

| Time | Repeat^#^ | *osarf12* KO#1 | *osarf12* KO#5 | *osarf12* KO#6 | WT(ZH11) |
| --- | --- | --- | --- | --- | --- |
| 1 wpi | R1 | 0 | 0 | 1 | 0 |
|  | R2 | 0 | 0 | 0 | 0 |
|  | R3 | 1 | 1 | 0 | 0 |
| 2 wpi | R1 | 3 | 3 | 2 | 1 |
|  | R2 | 4 | 5 | 3 | 1 |
|  | R3 | 2 | 4 | 1 | 0 |
| 3 wpi | R1 | 5 | 6 | 5 | 2 |
|  | R2 | 5 | 7 | 6 | 3 |
|  | R3 | 4 | 8 | 6 | 3 |
| 4 wpi | R1 | 7 | 8 | 9 | 5 |
|  | R2 | 6 | 9 | 10 | 5 |
|  | R3 | 7 | 10 | 10 | 4 |
| 5 wpi | R1 | 10 | 13 | 12 | 7 |
|  | R2 | 11 | 14 | 11 | 8 |
|  | R3 | 9 | 12 | 13 | 7 |
| 6 wpi | R1 | 14 | 15 | 14 | 9 |
|  | R2 | 13 | 14 | 15 | 11 |
|  | R3 | 13 | 15 | 16 | 10 |
| 7 wpi | R1 | 19 | 17 | 17 | 13 |
|  | R2 | 18 | 18 | 16 | 13 |
|  | R3 | 19 | 18 | 18 | 12 |
| 8 wpi | R1 | 22 | 21 | 22 | 16 |
|  | R2 | 23 | 22 | 21 | 17 |
|  | R3 | 22 | 20 | 21 | 15 |

^#^ For each repeat, 25 seedlings were inoculated with viruliferous leafhopper.
