# Supplementary material for: Auxin response factors (ARFs) differentially regulate rice antiviral immune response against rice dwarf virus
Source: PLoS Pathog. 2020 Dec 2;16(12):e1009118. doi: 10.1371/journal.ppat.1009118 (PMC7735678; doi:10.1371/journal.ppat.1009118)
Supplement: S3 Table — (DOCX) [file ppat.1009118.s017.docx]

**S8 Table. Constructs list.**

| **Use** | **Construct** | **Primers** | **Template** | **Plasmid** | **Cloning method** |
| --- | --- | --- | --- | --- | --- |
| Yeast two-hybrid | BD-OsARF1 | OsARF1F/OsARF1R | Rice cDNA | pGBKT7 | Gateway |
|  | BD-OsARF2 | OsARF2F/OsARF2R | Rice cDNA | pGBKT7 | Gateway |
|  | BD-OsARF3 | OsARF3F/OsARF3R | Rice cDNA | pGBKT7 | Gateway |
|  | BD-OsARF4 | OsARF4F/OsARF4R | Rice cDNA | pGBKT7 | Gateway |
|  | BD-OsARF5 | OsARF5F/OsARF5R | Rice cDNA | pGBKT7 | Gateway |
|  | BD-OsARF6 | OsARF6F/OsARF6R | Rice cDNA | pGBKT7 | Gateway |
|  | BD-OsARF7 | OsARF7F/OsARF7R | Rice cDNA | pGBKT7 | Gateway |
|  | BD-OsARF8 | OsARF8F/OsARF8R | Rice cDNA | pGBKT7 | Gateway |
|  | BD-OsARF9 | OsARF9F/OsARF9R | Rice cDNA | pGBKT7 | Gateway |
|  | BD-OsARF10 | OsARF10F/OsARF10R | Rice cDNA | pGBKT7 | Gateway |
|  | BD-OsARF11 | OsARF11F/OsARF11R | Rice cDNA | pGBKT7 | Gateway |
|  | BD-OsARF12 | OsARF12F/OsARF12R | Rice cDNA | pGBKT7 | Gateway |
|  | BD-OsARF13 | OsARF13F/OsARF13R | Rice cDNA | pGBKT7 | Gateway |
|  | BD-OsARF14 | OsARF14F/OsARF14R | Rice cDNA | pGBKT7 | Gateway |
|  | BD-OsARF15 | OsARF15F/OsARF15R | Rice cDNA | pGBKT7 | Gateway |
|  | BD-OsARF16 | OsARF16F/OsARF16R | Rice cDNA | pGBKT7 | Gateway |
|  | BD-OsARF17 | OsARF17F/OsARF17R | Rice cDNA | pGBKT7 | Gateway |
|  | BD-OsARF18 | OsARF18F/OsARF18R | Rice cDNA | pGBKT7 | Gateway |
|  | BD-OsARF19 | OsARF19F/OsARF19R | Rice cDNA | pGBKT7 | Gateway |
|  | BD-OsARF20 | OsARF20F/OsARF20R | Rice cDNA | pGBKT7 | Gateway |
|  | BD-OsARF21 | OsARF21F/OsARF21R | Rice cDNA | pGBKT7 | Gateway |
|  | BD-OsARF22 | OsARF22F/OsARF22R | Rice cDNA | pGBKT7 | Gateway |
|  | BD-OsARF23 | OsARF23F/OsARF23R | Rice cDNA | pGBKT7 | Gateway |
|  | BD-OsARF24 | OsARF24F/OsARF24R | Rice cDNA | pGBKT7 | Gateway |
|  | BD-OsARF25 | OsARF25F/OsARF25R | Rice cDNA | pGBKT7 | Gateway |
|  | AD-OsARF1 | OsARF1F/OsARF1R | pGBKT7-OsARF1 | pGADT7 | Gateway |
|  | AD-OsARF2 | OsARF2F/OsARF2R | pGBKT7-OsARF2 | pGADT7 | Gateway |
|  | AD-OsARF3 | OsARF3F/OsARF3R | pGBKT7-OsARF3 | pGADT7 | Gateway |
|  | AD-OsARF4 | OsARF4F/OsARF4R | pGBKT7-OsARF4 | pGADT7 | Gateway |
|  | AD-OsARF5 | OsARF5F/OsARF5R | pGBKT7-OsARF5 | pGADT7 | Gateway |
|  | AD-OsARF6 | OsARF6F/OsARF6R | pGBKT7-OsARF6 | pGADT7 | Gateway |
|  | AD-OsARF7 | OsARF7F/OsARF7R | pGBKT7-OsARF7 | pGADT7 | Gateway |
|  | AD-OsARF8 | OsARF8F/OsARF8R | pGBKT7-OsARF8 | pGADT7 | Gateway |
|  | AD-OsARF9 | OsARF9F/OsARF9R | pGBKT7-OsARF9 | pGADT7 | Gateway |
|  | AD-OsARF10 | OsARF10F/OsARF10R | pGBKT7-OsARF10 | pGADT7 | Gateway |
|  | AD-OsARF11 | OsARF11F/OsARF11R | pGBKT7-OsARF11 | pGADT7 | Gateway |
|  | AD-OsARF12 | OsARF12F/OsARF12R | pGBKT7-OsARF12 | pGADT7 | Gateway |
|  | AD-OsARF13 | OsARF13F/OsARF13R | pGBKT7-OsARF13 | pGADT7 | Gateway |
|  | AD-OsARF14 | OsARF14F/OsARF14R | pGBKT7-OsARF14 | pGADT7 | Gateway |
|  | AD-OsARF15 | OsARF15F/OsARF15R | pGBKT7-OsARF15 | pGADT7 | Gateway |
|  | AD-OsARF16 | OsARF16F/OsARF16R | pGBKT7-OsARF16 | pGADT7 | Gateway |
|  | AD-OsARF17 | OsARF17F/OsARF17R | pGBKT7-OsARF17 | pGADT7 | Gateway |
|  | AD-OsARF18 | OsARF18/OsARF18R | pGBKT7-OsARF18 | pGADT7 | Gateway |
|  | AD-OsARF19 | OsARF19F/OsARF19R | pGBKT7-OsARF19 | pGADT7 | Gateway |
|  | AD-OsARF20 | OsARF20F/OsARF20R | pGBKT7-OsARF20 | pGADT7 | Gateway |
|  | AD-OsARF21 | OsARF21F/OsARF21R | pGBKT7-OsARF21 | pGADT7 | Gateway |
|  | AD-OsARF22 | OsARF22F/OsARF22R | pGBKT7-OsARF22 | pGADT7 | Gateway |
|  | AD-OsARF23 | OsARF23F/OsARF23R | pGBKT7-OsARF23 | pGADT7 | Gateway |
|  | AD-OsARF24 | OsARF24F/OsARF24R | pGBKT7-OsARF24 | pGADT7 | Gateway |
|  | AD-OsARF25 | OsARF25F/OsARF25R | pGBKT7-OsARF25 | pGADT7 | Gateway |
| Transient expression in tobacco | pWM101:HA-OsARF5 | HAOsARF5F-KpnI/OsARF5R-SalI | pGBKT7-OsARF5 | pWM101 | KpnI/SalI |
|  | pWM101:HA-OsARF6 | HAOsARF6F-KpnI/OsARF6R-SmaI | pGBKT7-OsARF6 | pWM101 | KpnI/SmaI |
|  | pWM101:HA-OsARF11 | HAOsARF11F-KpnI/OsARF11R- BamHI | pGBKT7-OsARF11 | pWM101 | KpnI/BamHI |
|  | pWM101:HA-OsARF12 | HAOsARF12F-KpnI/OsARF12R- BamHI | pGBKT7-OsARF12 | pWM101 | KpnI/BamHI |
|  | pWM101:HA-OsARF16 | HAOsARF16F-KpnI/OsARF16R- SmaI | pGBKT7-OsARF16 | pWM101 | KpnI/SmaI |
|  | pWM101:HA-OsARF17 | HAOsARF17F-KpnI/OsARF17R- SmaI | pGBKT7-OsARF17 | pWM101 | KpnI/SmaI |
|  | pWM101:HA-OsARF19 | HAOsARF19F-KpnI/OsARF19R- SalI | pGBKT7-OsARF19 | pWM101 | KpnI/SalI |
|  | pWM101:HA-OsARF21 | HAOsARF21F- SalI/OsARF21R- SalI | pGBKT7-OsARF21 | pWM101 | SalI |
|  | pWM101:HA-OsARF25 | HAOsARF25F-KpnI/OsARF25R- SmaI | pGBKT7-OsARF25 | pWM101 | KpnI/SmaI |
| LCI assay | cLUC-OsARF5 | OsARF5cLUCF-KpnI/OsARF5cLUCR-SalI | pGBKT7-OsARF5 | pCambia 1300 | KpnI/SalI |
|  | cLUC-OsARF6 | OsARF6cLUCF-KpnI/OsARF6cLUCR-KpnI | pGBKT7-OsARF6 | pCambia 1300 | KpnI |
|  | cLUC-OsARF11 | OsARF11cLUCF-KpnI/OsARF11cLUCR-KpnI | pGBKT7-OsARF11 | pCambia 1300 | KpnI |
|  | cLUC-OsARF12 | OsARF12cLUCF-KpnI/OsARF12cLUCR-KpnI | pGBKT7-OsARF12 | pCambia 1300 | KpnI |
|  | cLUC-OsARF16 | OsARF16cLUCF-KpnI/OsARF16cLUCR-SalI | pGBKT7-OsARF16 | pCambia 1300 | KpnI/SalI |
|  | cLUC-OsARF17 | OsARF17cLUCF-KpnI/OsARF17cLUCR-BamHI | pGBKT7-OsARF17 | pCambia 1300 | KpnI/BamHI |
|  | cLUC-OsARF19 | OsARF19cLUCF-KpnI/OsARF19cLUCR-SalI | pGBKT7-OsARF19 | pCambia 1300 | KpnI/SalI |
|  | cLUC-OsARF21 | OsARF21cLUCF-KpnI/OsARF21cLUCR-SalI | pGBKT7-OsARF21 | pCambia 1300 | KpnI/SalI |
|  | cLUC-OsARF25 | OsARF25cLUCF-KpnI/OsARF25cLUCR-BamHI | pGBKT7-OsARF25 | pCambia 1300 | KpnI/BamHI |
| Rice transgene | Actin-HA-OsARF12 | HA-OsARF12F-XbaI/OsARF12R-XbaI | pGBKT7-OsARF12 | pCambia 2300 | XbaI |
| Protein purification | GST-OsARF12DBD | OsARF12DBDF/OsARF12DBDR | pGBKT7-OsARF12 | pGEX-4T-1 | EcoR1/SalI |
