# Supplementary material for: Auxin response factors (ARFs) differentially regulate rice antiviral immune response against rice dwarf virus
Source: PLoS Pathog. 2020 Dec 2;16(12):e1009118. doi: 10.1371/journal.ppat.1009118 (PMC7735678; doi:10.1371/journal.ppat.1009118)
Supplement: S4 Table — (DOCX) [file ppat.1009118.s018.docx]

**S4** **Table. Primers list.**

| **Primer name** | **Primer Sequence 5’-3’** | **Use** |
| --- | --- | --- |
| HAOsARF5F-KpnI | GAGGTACCATGTACCCATACGATGTTCCAGATTACGCGAAGCAGCAGCAGACGCCGGCGAG | Plasmids constructions |
| OsARF5R-SalI | GCGTCGACTCATTCGAATTGATCATACGGGCCGA |  |
| HAOsARF6F-KpnI | GCGGTACCATGTACCCATACGATGTTCCAGATTACGCGAAGCTCTCGCCGTCGGC |  |
| OsARF6R-SmaI | CGCCCGGGTCAGAACTCAACTGAGCCCACC |  |
| HAOsARF11F-KpnI | GCGGTACCATGTACCCATACGATGTTCCAGATTACGCGGCGTCCTCGCAG |  |
| OsARF11R-BamHI | GCGGATCCCTAAGCTGCTTGAATACAGTC |  |
| HAOsARF12F-KpnI | GCGGTACCATGTACCCATACGATGTTCCAGATTACGCGAGCTCGTCGTTCGGCG |  |
| OsARF12R-BamHI | GCGGATCCTCAGGACAGATACCGTGGATC |  |
| HAOsARF16F-KpnI | GCGGTACCATGTACCCATACGATGTTCCAGATTACGCGAAGGATCAGGGATCATC |  |
| OsARF16R-SmaI | CGCCCGGGTCAACCTCTCCATGCATTTGCATC |  |
| HAOsARF17F-KpnI | GCGGTACCATGTACCCATACGATGTTCCAGATTACGCGAGGCTTTCGTCGTCGTC |  |
| OsARF17R-SmaI | CGCCCGGGTCAGAATTCAACTGAGCCGACAG |  |
| HAOsARF19F-KpnI | GAGGTACCATGTACCCATACGATGTTCCAGATTACGCGATGAAGCAGGCGCAGCAG |  |
| OsARF19R-SalI | GCGTCGACTCATTCGAATTGTTCATATG AACCATTGGAAGGG |  |
| HAOsARF21F-SalI | GAGTCGACATGTACCCATACGATGTTCCAGATTACGCGCGAAAGGACGCAGATGCGC |  |
| OsARF21R-SalI | GCGTCGAC CTAGTCGCCG GTTATCCTCC |  |
| HAOsARF25F-KpnI | GCGGTACCATGTACCCATACGATGTTCCAGATTACGCGAAGCTCTCTCCGCCGGC |  |
| OsARF25R-SmaI | CGCCCGGGTCAGTAGTCCAGAGGCGCTAC |  |
| OsARF5cLUCF-KpnI | GAGGTACCATGAAGCAGCAGCAGACG |  |
| OsARF5cLUCR-SalI | GCGTCGACTCATTCGAATTGATCATACGG |  |
| OsARF6cLUCF-KpnI | GCGGTACCATGAAGCTCTCGGCCGTCGGC |  |
| OsARF6cLUCR-KpnI | CGGGTACCTCAGAACTCAACTGAGCCCACC |  |
| OsARF11cLUCF-KpnI | GCGGTACCATGGCGTCCTCGCAGGAG |  |
| OsARF11cLUCR-KpnI | GCGGTACCCTAAGCTGCTTGAATACAGTC |  |
| OsARF12cLUCF-KpnI | GCGGTACCATGAGCTCGTCGTCGGCG |  |
| OsARF12cLUCR-KpnI | GCGGATCCTCAGGACAGATACCGTGGATC |  |
| OsARF16cLUCF-KpnI | GCGGTACCATGAAGGATCAGGGATCATC |  |
| OsARF16cLUCR-SalI | GCGTCGACACCTCTCCATGCATTTGCATC |  |
| OsARF17cLUCF-KpnI | GCGGTACCATGATGAGGCTTTCGTCGTCGTC |  |
| OsARF17cLUCR-BamHI | CGGGTACCTCAGAATTCAACTGAGCCGACAGATG |  |
| OsARF19cLUCF-KpnI | GAGGTACC ATGATGAAGCAGGCGCAGCAG |  |
| OsARF19cLUCR-SalI | GCGTCGACTTCGAATTGTTCATATGAACCATTGGAAGGG |  |
| OsARF25cLUCF-KpnI | GCGGTACCATGAAGCTCTCTCCGCCGGC |  |
| OsARF25cLUCR-BamHI | CGGGATCCTCAGTAGTCCAGAGGCGCTAC |  |
| Tos17-BP | AGGTTGCAAGTTAGTTAAGA |  |
| *osarf12*LP | GCAGTTCTCCCAAGTCCAAC |  |
| *osarf12*RP | TTAACTTGGCCGTTTTGAGG |  |
| *osarf11*LP | TTACTCCTACGGACCTTGCG |  |
| *osarf11*RP | TCAGCTTGCAGTATGGATGC |  |
| *osarf16*LP | TGAATGTGACACATTGCAGAAC |  |
| *osarf16*RP | CACCAGCACAAGAGCTCATC |  |
| HAOsARF12F-XbaI | GCTCTAGAATGTACCCATACGATGTTCCAGATTACGCGAGCTCGTCGTCGGCG |  |
| OsARF12R-XbaI | GCTCTAGATCAGGACAGATACCGTGGATC |  |
| OsARF12DBDF | GCGAATTCCCCGCGGAGATGGGGATAAT |  |
| OsARF12DBDR | GCGTCGACAACAGAGGAAGGCATCACAGT |  |
| q-OsIAA10-F | GGTTGCTGGATGGGTGAAGG | Quantitative Real-time PCR |
| q-OsIAA10-R | CCTGTCCTCGTAGGTGAGCTGG |  |
| q-OsGH3.2-F | TCATGCCCGTCATGAACTTG |  |
| q-OsGH3.2-R | TCGTCTCCGACTTGATGAACAG |  |
| q-OsWRKY13-F | TTTGGGAAAGCGTTGATTAGT |  |
| q-OsWRKY13-R | GCGCACACACACTCCAACTC |  |
| q-S2-F | GCTATACACATCATCGCCGTGGTGT |  |
| q-S2-R | AACTTTGCTTCGGTGGTTGCCCCTG |  |
| q-S8-F | GATATACCCTATTCTGAACCTATTG |  |
| q-S8-R | GTTATCGAGTTCAATGTATAGTAGG |  |
| q-OsEF1a-F | ACATTGCCGTCAAGTTTGCTG |  |
| q-OsEF1a-R | AACAGCCACCGTTTGCCTC |  |
| q-S11-F | GGTAAATGAGTGGAACATTACCCTTG |  |
| q-S11-R | AAATCCGTTTCAGAATTTGAGGAAT |  |
| q-YUCCA6-F | ATGGCAGCACTGAAGAAT |  |
| q-YUCCA6-R | TTCCACGCATTTCCCTTT |  |
| q-TRPC-F | GGCATCAATAACCGAAGT |  |
| q-TRPC-R | TCACCAACAACCAGAATC |  |
| q-YUCCA8-F | CATGTTCTACCGAGAGAG |  |
| q-YUCCA8-R | AACCTATCCACCATCTTG |  |
| q-AAO2-F | CTCAGCCATCAATAGAAG |  |
| q-AAO2-R | ATCCGACAATATCTCAATG |  |
| q-AAO1-F | CTGTATGGTCGTGTATAG |  |
| q-AAO1-R | GGACATTATGACAAGGTA |  |
| q-TSA1-F | CGCATTCATTCCATTCATC |  |
| q-TSA1-R | AACCACAAGCATCAAGAA |  |
| q-TRP1-F | TTCAATATCCTTGGTCCTCTC |  |
| q-TRP1-R | GGTAACTATGTTCTCGTGGTA |  |
| q-TRP4-F | AGAGCATCATAACAACAGA |  |
| q-TRP4-R | CACGGTAAGCAATTCAAG |  |
| q-OsJAZ12-F  q-OsJAZ12-R | ATCTGCCCGGTTTAGAGGAG  GGGCCAAAGAAATCTCAAAC |  |
| q-OsPR10-F | CCCTGCCGAATACGCCTAA |  |
| q-OsPR10-R | CTCAAACGCCACGAGAATTTG |  |
| q-OsWRKY45-F | CGGGTAAAACGATCGAAAGA |  |
| q-OsWRKY45-R | TTTCGAAAGCGGAAGAACAG |  |
| q-OsPR2-F | TTCAACGAGAACCAGAAG |  |
| q-OsPR2-R | TTAGAAATTGATGGAGTATGC |  |
| q-OsPR1a-F | CGTCTTCATCACCTGCAACTACTC |  |
| q-OsPR1a-R | CATGCATAAACACGTAGCATAGCA |  |
| q-OsICS1-F | TATGGTGCTATCCGCTTCGAT |  |
| q-OsICS1-R | CGAGAACCGAGCTCTCTTCAA |  |
| q-OsPAD4-F | GCCAGCTCCCCTACGACTTC |  |
| q-OsPAD4-R | CGTGTGCGGTGTAGGTTGTT |  |
| Actin-F-RT | ATGGTCAAGGCTGGGTTT | RT-PCR and Probe Synthesis |
| Actin-R-RT | CGACATACATAGCTGGCACA |  |
| OsARF12F-RT | CAGCCTGTAGCTAGTGAGCAG |  |
| OsARF12R-RT | TCCCAAGGGTCGTCTCCAAG |  |
| OsARF11F-RT | ACACCACTTCATGCTCTCAACC |  |
| OsARF11R-RT | CTAAGCTGCTTGAATACAGTCGT |  |
| OsARF16F-RT | GCTGACGGCGAGCGACACAAG |  |
| OsARF16R-RT | GCGCACTGGATCCAAGTCACC |  |
| S11-F | ATGAGTGGAACATTACCCTTGG |  |
| S11-R | TTACTTACGCTTTGATTTGCG |  |
| S2-F | ATAATGCCATATCTCTCACCATTGC |  |
| S2-R | TAAGGGCGAGCCTCAACGTCATACG |  |
| S8-F | GATATACCCTATTCTGAACCTATTG |  |
| S8-R | GTTATCGAGTTCAATGTATAGTAGG |  |
| ChIP-OsWRKY13-F | GCGTACATACACGTTCATGTG |  |
| ChIP- OsWRKY13-R | CCTTGGCTACATGCGTGTTTG |  |
| ChIP-Actin-F | TGGCATCTCTCAGCACATTC | ChIP-qRT-PCR and EMSA Probe Synthesis |
| ChIP-Actin-R | GGCAAGCAACATTGTAAGCA |  |
| EMSA-OsWRKY13-F | TGGTTCGTGATTAAGGGTTTGGTTACACCGTGTCCCGCTCGCGGCACGGATAGGCTGCTTAATTCTCTTT |  |
| EMSA-OsWRKY13-R | AAAGAGAATTAAGCAGCCTATCCGTGAGCGGGACACGGTGTAACCAAACCCTTAATCACGAACCA |  |
| EMSA-OsWRKY13-Cold-F | GTTACACCGTGTCCCGCTCACGGAT |  |
| EMSA-OsWRKY13-Cold-R | ATCCGTGAGCGGGACACGGTGTAAC |  |
| EMSA-OsWRKY13-mCold-F | GTTACACCGAAAAAAGCTCACGGAT |  |
| EMSA-OsWRKY13-mCold-R | ATCCGTGAGCTTTTTTCGGTGTAAC |  |
